# Nutrient History Affects the Response and Resilience of the Tropical Seagrass *Halophila stipulacea* to Further Enrichment in Its Native Habitat

**DOI:** 10.3389/fpls.2021.678341

**Published:** 2021-08-05

**Authors:** Stephanie B. Helber, Gidon Winters, Marleen Stuhr, E. F. Belshe, Stefanie Bröhl, Michael Schmid, Hauke Reuter, Mirta Teichberg

**Affiliations:** ^1^Leibniz Centre for Tropical Marine Research (ZMT) GmbH, Bremen, Germany; ^2^Institute for Chemistry and Biology of the Marine Environment (ICBM), Oldenburg, Germany; ^3^The Dead Sea and Arava Science Center (ADSSC), Jerusalem, Israel; ^4^Ben-Gurion University of the Negev, Eilat, Israel; ^5^Tropical Coral Ecophysiology, Interuniversity Institute for Marine Sciences - Eilat (IUI), Eilat, Israel; ^6^The Mina and Everard Goodman Faculty of Life Sciences, Bar-Ilan University (BIU), Ramat Gan, Israel; ^7^Faculty for Biology and Chemistry, University of Bremen, Bremen, Germany

**Keywords:** Red Sea, global warming, eutrophication, seagrass degradation, warning indicator, pollution, multiple stressors, Gulf of Aqaba (Eilat)

## Abstract

Eutrophication is one of the main threats to seagrass meadows, but there is limited knowledge on the interactive effects of nutrients under a changing climate, particularly for tropical seagrass species. This study aimed to detect the onset of stress in the tropical seagrass, *Halophila stipulacea*, by investigating the effect of *in situ* nutrient addition during an unusually warm summer over a 6-month period. We measured a suite of different morphological and biochemical community metrics and individual plant traits from two different sites with contrasting levels of eutrophication history before and after *in situ* fertilization in the Gulf of Aqaba. Nutrient stress combined with summer temperatures that surpassed the threshold for optimal growth negatively affected seagrass plants from South Beach (SB), an oligotrophic marine protected area, while *H. stipulacea* populations from North Beach (NB), a eutrophic and anthropogenically impacted area, benefited from the additional nutrient input. Lower aboveground (AG) and belowground (BG) biomass, reduced Leaf Area Index (LAI), smaller internodal distances, high sexual reproductive effort and the increasing occurrence of apical shoots in seagrasses from SB sites indicated that the plants were under stress and not growing under optimal conditions. Moreover, AG and BG biomass and internodal distances decreased further with the addition of fertilizer in SB sites. Results presented here highlight the fact that *H. stipulacea* is one of the most tolerant and plastic seagrass species. Our study further demonstrates that the effects of fertilization differ significantly between meadows that are growing exposed to different levels of anthropogenic pressures. Thus, the meadow’s “history” affects it resilience and response to further stress. Our results suggest that monitoring efforts on *H. stipulacea* populations in its native range should focus especially on carbohydrate reserves in leaves and rhizomes, LAI, internodal length and percentage of apical shoots as suitable warning indicators for nutrient stress in this seagrass species to minimize future impacts on these valuable ecosystems.

## Introduction

Seagrass meadows are becoming increasingly threatened, as their distribution along coastal areas makes them especially vulnerable toward local anthropogenic pressures (Orth et al., 2006; Wilkinson and Salvat, 2012). Seagrasses are disappearing four times faster than tropical rainforests (Masakazu et al., 2019), causing great concern considering the biodiversity associated with seagrass meadows, alongside the wide range of valuable ecosystem services and functions that these meadows provide. In addition to being vital for adjacent ecosystems (e.g., coral reefs), many of these services and functions also have important impacts on local economies and human health. Tropical seagrasses benefit neighboring coral reefs by increasing water quality, decreasing sediment resuspension and particulate matter loading (Orth et al., 2006; Gillis et al., 2014), mitigating the effects of ocean acidification (Unsworth et al., 2012; Bergstrom et al., 2019) and by reducing pathogen concentrations that would also have negative impacts on humans (Lamb et al., 2017).

*Halophila stipulacea* (Forsskål) Ascherson is a tropical seagrass species native to the Indian Ocean, Red Sea and Persian Gulf (Lipkin, 1975). It invaded the eastern Mediterranean through the Suez Canal some 150 years ago (Lipkin, 1975; Sghaier et al., 2011), but has also expanded into the Caribbean, where it outcompetes the native seagrass species *Syringodium filiforme*, *Halodule wrightii*, and *Halophila decipiens* (Ruiz and Ballantine, 2004; Willette and Ambrose, 2012; Willette et al., 2014; Smulders et al., 2017; Winters et al., 2020). Contrary to other seagrass species, *H. stipulacea* is able to survive under a broad range of temperatures (11 to 38°C), shows a high photo-physiological plasticity and has been found to thrive under eutrophic conditions, even in sulfidic sediments (Sharon et al., 2009; van Tussenbroek et al., 2016; Rotini et al., 2017; Wesselmann et al., 2020; reviewed by Winters et al., 2020). The ability of *H. stipulacea* to rapidly acclimate to a wide range of environmental conditions paired with its fast growth rates, leaf turnover and high fragment dispersal are possible reasons for its worldwide colonization success and, hence, sparked interests in its population dynamics as well as its resilience toward anthropogenic pressures (Weatherall et al., 2016; O’Brien et al., 2018b).

Research efforts in the Gulf of Aqaba (GoA), northern Red Sea, have mainly focused on local coral reefs (e.g., Rinkevich, 2005; Fine et al., 2013, 2019), even though an extensive area of approximately 707,000 m^2^ along the coastline is covered by seagrass meadows (Winters et al., 2017). *H. stipulacea* is the most dominant and widespread seagrass species in the GoA (Winters et al., 2017), where it forms large, discontinuous meadows in both shallow and upper mesophotic zones (1–51 m depth) (Sharon et al., 2011; Kramer et al., 2019). These seagrass meadows along the coastline of Eilat, Israel, provide important ecosystem services that are estimated to exceed US$ 2,000,000 per year (Winters et al., 2017). However, the importance of these ecosystems is still not fully recognized, not only by scientists but also by policy makers and local stakeholders (Winters et al., 2017, 2020).

The semi- enclosed nature of the Red Sea makes it particularly vulnerable towards eutrophication and global warming. It has been shown that annual sea surface temperatures (SSTs) in the northern GoA are increasing at rates of 0.254 ± 0.058°C decade^–1^ (Fine et al., 2013; Nguyen et al., 2020), slightly faster than the global coastal SST trend of 0.17 ± 0.11°C decade^–1^ (Liao et al., 2015). The cities of Eilat and Aqaba, located on the northernmost part of the GoA, are the largest, fast growing population centers in the region, both of which attract millions of tourists annually. In both these cities, adjacent coastal ecosystems face numerous threats due to significant infrastructure development as one of the Red Sea’s major tourist destinations (Fine et al., 2019). In addition to global threats of ocean warming and acidification, the main regional threats to local marine ecosystems include enhanced nutrient inputs (i.e., eutrophication) from sewage, mariculture and agricultural runoff (Loya, 2004; Winters et al., 2017). There is no natural terrestrial run off into the Red Sea since it is surrounded by deserts and only nutrient- depleted Red Sea surface water can enter the GoA through the Straits of Tiran making the Red Sea world famous for its oligotrophic, clear waters (Stambler, 2005; Wurgaft et al., 2016). Due to the GoA being a semi- enclosed ocean body, its waters are characterized by a particularly long residence time of 3–8 years (Loya and Kramarsky-Winter, 2003; Winters et al., 2017). This long residence time means that the effects of land-associated sources of pollution, such as high nutrient inputs from poorly or non-treated sewage as well as municipal discharge, mari- and agricultural runoffs, and severe flash flood events, are intensified several folds. These local anthropogenic stressors may, in combination with global warming, affect seagrass ecosystems even more dramatically than previously predicted by experiments that have been conducted with only one stressor in laboratory facilities without including the whole seagrass ecosystem (Egea et al., 2018; Mvungi and Pillay, 2019; Nguyen et al., 2020; Pazzaglia et al., 2020). Studies investigating the effect of multiple stressors on *H. stipulacea* meadows *in situ* within its native range are particularly scarce, yet these studies will be crucial if we want to understand how environmental and anthropogenic pressures affect the state and population dynamics of this seagrass species. Moreover, multiple stressor studies on *H. stipulacea* in its native range will inform us how to improve management efforts for its conservation in the northern GoA since establishing marine protected areas (MPAs) alone has already been shown to be insufficient for protecting seagrass meadows (Eklöf et al., 2009; Quiros et al., 2017). Identifying how different stressors impact these ecosystems is of fundamental importance if we are to take timely targeted management actions.

The aim of our study was to identify warning indicators for nutrient stress in *H. stipulacea* seagrass meadows growing in both anthropogenically low and high impacted sites. For this we compared the effects of nutrient history on seagrass responses to *in situ* nutrient enrichment, created by using fertilizer addition over 6 months, capturing as well the high summer temperature peak. Signs of stress were investigated on individual plant as well as on the population level by measuring structural community metrics (% cover, shoot density, biomass) and morphological (leaf width, length, leaf area, leaf area index, % apical shoots, no. of leaves, internodal distance) and biochemical (tissue nutrient content and carbohydrates) plant traits identified as potential stress response indicators (Roca et al., 2016). This study is part 2 of a parallel study on *Posidonia oceanica* in the Mediterranean (Helber et al., 2021). Through both of these studies, we aim to compare responses of *H. stipulacea*, a fast-growing, small-bodied seagrass, to *P. oceanica*, a slow-growing, large-bodied seagrass, in order to better understand how these very different seagrasses may respond under future nutrient and climate scenarios and potentially interact in areas where *H. stipulacea* occurs within *P. oceanica* meadows (Beca-Carretero et al., 2020b).

## Materials and Methods

### Study Sites

**FIGURE 1:**
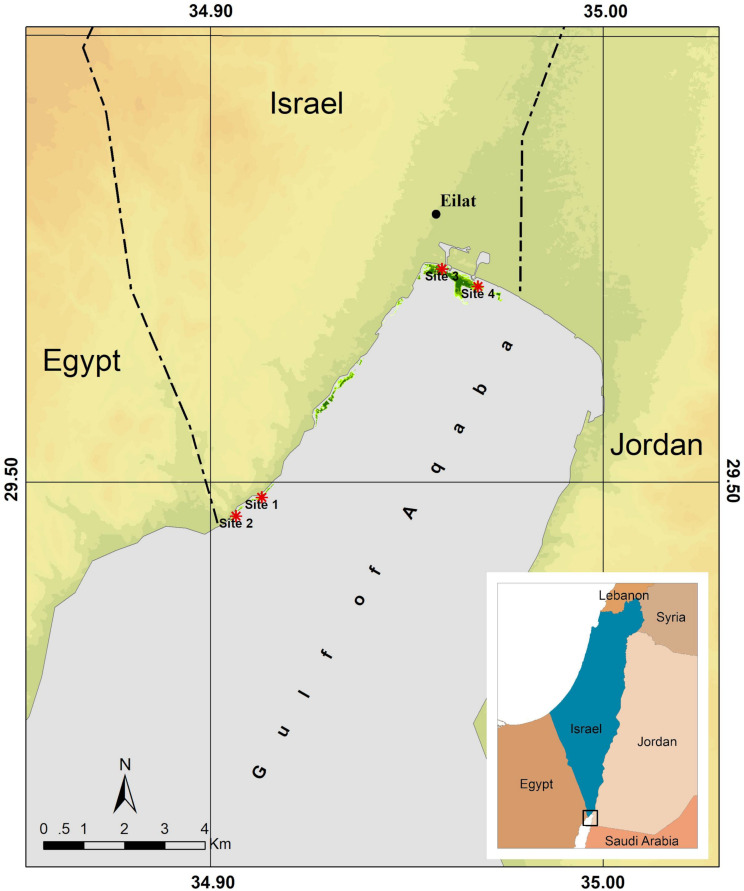
Overview of the northern Gulf of Aqaba with our study sites. Shown here are the study sites within the marine protected South Beach area (S1 and S2) and the study sites at the North Beach area (S3 and S4), which are exposed to anthropogenic pressures. S3 is close to a marina and S4 is close to an outlet draining local agricultural fields and land-based fish farms (Winters et al., 2017). Seagrass meadow extent is indicated in green (adapted from Winters et al., 2017).

The response of *H. stipulacea* populations to thermal and nutrient stress were compared between two sites that are already heavily exposed to anthropogenic pressures (North Beach – NB) and two sites in a marine protected area with relatively low anthropogenic pressures (South Beach – SB) along the western coast of the northern Gulf of Aqaba (Eilat, Israel; Figure 1). The South Beach area is characterized by a steep slope, gravel sized sediment and a high cover of neighboring corals. In terms of light penetration, this site has been shown to have a low photosynthetic active radiation (PAR) diffuse attenuation coefficient (*K*_d_ PAR), entailing a relatively high Secchi disk depth and relatively small seagrass leaves (Mejia et al., 2016; Beca-Carretero et al., 2020a). Lastly, this site falls within a local MPA with little tourist infrastructure, no buildings at the beaches, but is a popular diving spot. In contrast, seagrass meadows on the North Beach are in close proximity to a dense strip of hotels and a marina. Extensive boating activities occur right over the meadows. The North Beach site is characterized by a small slope and fine, muddy sediment, and hardly any corals. The light attenuation (*K*_d_) determined by measurements of PAR at the water surface and sediment depth, is much higher than in the South Beach site (Beca-Carretero et al., 2020a), and secchi disk depth has been found to be much shallower due to less light penetrating the water, and the leaves of local seagrass plants are much larger (Mejia et al., 2016; Supplementary Figures 1, 2). Although removed some 10 years ago, gilthead sea breams (*Sparus aurata*) were cultivated in cages in this area for approximately 20 years, which led to nutrient enrichment of the water column and underlying sediments, and a die-off of the underlying seagrass meadows (Oron et al., 2014). It was estimated that the North Beach area received annually more than 250 tons of N and 50 tons of P as a result of the fish farming activities (fish excretion and undigested feed pellets) by the end of the 1990s and beginning of the 2000s (Lazar et al., 2008). This area is also exposed to sewage run-offs, the effects of flash floods, and the Kinnet Canal that bring nutrients into the local meadows (Mejia et al., 2016; Winters et al., 2017). Sewage run off contributed to approximately 150 tons of N per year during the 1980s and 1990s, but almost stopped in 1995 (Lazar et al., 2008). Recent stable nitrogen isotope work by Beca-Carretero et al. (2020a) found inputs of anthropogenic-origin nutrients (δ^15^N) and higher levels of NH_4_^+^ in seawater in the NB than in the SB alongside a higher annual signal of δ^15^N in *H. stipulacea* plants growing in the more impacted site (NB) compared with the more pristine site (SB). These results indicate a potential anthropogenic input of N in the seawater, which is reflected in the plant content in the impacted site (NB), and together with the light measurements and leaf morphometrics (detailed above; Mejia et al., 2016; Beca-Carretero et al., 2020a) demonstrate a difference between the two sites.

### Experimental Set-Up

**FIGURE 2:**
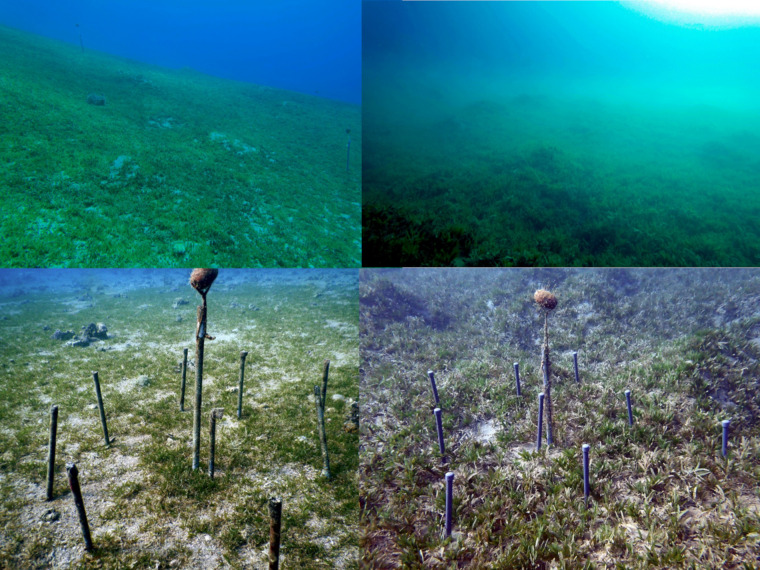
Overview of the study sites within the MPA South Beach **(Left side)** and the North Beach **(Right side)** exposed to anthropogenic pressures. The lower panel shows the set- up of the plots. Pictures: Upper panel, Dr. MiS; lower panel left, MaS; lower panel right Julia Cerutti.

Six circular plots with a diameter of 2m were established at each one of the four sites (S1–S4), at least 10 m apart from each other. Three of these plots were used as control, while the other three were enriched with slow-release fertilizer pellets (Osmocote^®^ Pro: 19% N – 3.9% P – 8.3% K, ICL Speciality Fertilizers). The fertilizer contained nitrogenous compounds that were composed of nitrate (6.3%), ammonia (8.2%) and urea (4.5%). Fertilizer pellets were filled in 0.5 m long PVC tubes, which were hammered approximately 20 cm into the sediment, delivering nutrients to the below-ground and above-ground tissues (Worm et al., 2000). Nine fertilizer filled PVC tubes were inserted in each nutrient treatment plot resulting in an addition of approximately 1,170 g of fertilizer per plot (Figure 2). All PVC tubes used in treatment and control plots were previously drilled throughout their entire length with 2mm holes to allow the flushing of seawater through the tubes into the surrounding environments.

From each of the plots at all four sites, we measured a number of target variables in July and December 2019 for assessing different levels of nutrient concentrations as well as biological organization, ranging from biochemical and morphological individual plant traits, to community level metrics as outlined below.

### Environmental Sampling

Water samples were taken with acid rinsed plastic syringes (approximately 30 mL) at around 10 m depth above the seagrass canopy of each plot on each site for nutrient analysis of the surrounding water. Porewater samples were taken by placing a syringe 5 cm deep into the sediment and carefully drawing out water from interstitial spaces of the sediment to determine porewater nutrient concentrations. Once on shore, water samples were immediately filtered into HDPE vials using sterile syringe filters (LABSOLUTE^®^; cellulose acetate; 0.45 μm pore size) and stored on ice. Samples were frozen at −20°C upon arrival to the laboratory on the same day of sampling and stored for further analyses. Nutrient measurements for samples taken in July were performed spectrophotometrically with a TECAN plate reader (Infinite 200 Pro microplate reader; Switzerland) according to Laskov et al. (2007). The detection limits were 0.08, 0.32, 0.7, and 0.022 μM for NO_2_^–^, NO_x_ (NO_3_^–^ and NO_2_^–^), NH_4_^+^, PO_4_^3–^, respectively. The NO_x_ (NO_3_^–^ and NO_2_^–^) and NH_4_^+^ concentrations sum up to dissolved inorganic nitrogen (DIN). The coefficient of variation was always <3.4%. The lower nutrient concentrations from samples taken during December 2019 were analyzed using a continuous flow injection analyzing system (Skalar SAN+++System). The detection limits were 0.043, 0.094, 0.290, and 0.042 μM for NO_2_^–^, NO_x_ (NO_3_^–^ and NO_2_^–^), NH_4_^+^, and PO_4_^3–^, respectively. The coefficient of variation was always <3.4%.

In addition to water samples taken within the plots, the water quality of the different sites was assessed by taking water samples directly above the seagrass canopy with a pre-rinsed 5-L Niskin (HDPE) bottle. Collected seawater was immediately stored in the dark on ice. Environmental parameters, such as salinity, temperature, oxygen and pH were measured directly after collection with a multi parameter probe (WTW Multiprobe). Moreover, temperature loggers (HOBO Water Temp Pro v2) were fixed to the main pole of plots 1 and 6 in the seagrass meadows (ca. 10 m water depth) to record the water temperatures hourly during the entire time of the experiment.

A defined volume of water was vacuum-filtered onto pre-combusted (5 h, 450°C) Whatman^®^ GF/F filters on return to the laboratories of the Interuniversity Institute for Marine Sciences in Eilat (IUI). Filters for chlorophyll *a* (Chl *a*) analysis were immediately stored at −80°C, filters for organic carbon (*C*_*org*_), total carbon (C) and nitrogen (N) content were frozen at −20°C until further analysis at the Leibniz Centre for Tropical Marine Research (ZMT) in Germany. Filters for suspended particulate matter (SPM) determination were weighed prior to filtering. Subsequently, filters were dried at 40°C for 48 h and the dried filters were weighed again. Filters for *C*_*org*_, C and N content were divided in 4 equal pieces and one quarter was used for each analysis. For Chl *a* analysis, filters were analyzed and extracted in 80°C hot ethanol, following Welshmeyer (1994). The supernatant was subsequently transferred into small vials and Chl *a* concentrations were determined with a TD10AU-Fluorometer (Turner Designs). The detection limit of this method is 0.002 μg l^–1^.

### Percent Cover

Percent cover was measured by randomly placing a 25 cm × 25 cm quadrat in each plot and taking a picture with an underwater camera. Pictures were analyzed with Coral Point Count for Excel (CPCe; Kohler and Gill, 2006). 100 points were randomly overlaid onto the images. Points intersecting with the occurrence of seagrasses were counted as “hit” and used to quantify the percentage of seagrass cover (0–100%).

### Shoot Density, Biomass, and Morphological Parameters

A biomass corer was used to sample above ground (AG) and below ground (BG) seagrass biomass in July and December 2019 at SB and NB sites. Samples were taken with an open- ended, standard PVC drainpipe with a diameter of 12.5 cm and a length of 0.5 m. In each plot, biomass cores were taken by twisting and hammering the corer approximately 10–15 cm into the sediment. The contents of the biomass cores were transferred underwater into diving mesh-bags and sediment was removed carefully underwater. Upon arrival at the laboratory facilities of the IUI in Eilat, biomass samples were carefully cleaned and all sand, sediment particles and epiphytes were carefully removed. Plant material from the core samples was separated into AG (leaves) and BG (rhizomes and roots) plant compartments. Shoot density was determined by counting the total number of shoots in each biomass core sample and then normalizing these counts per m^2^. Shoots that were examined for the presence of male or female reproductive structures were counted separately and the flowering percentage (no. of flowering shoots/total no. of shoots × 100) was calculated (Malm, 2006; Nguyen et al., 2018). AG and BG material was dried at 60°C for 48 h and then weighed to determine AG and BG biomasses (g DW m^–2^) and the above/below-ground biomass ratios (AG:BG).

Additionally, leaf and rhizome samples were taken from each biomass core to evaluate shoot morphology. Internodal distance of rhizome was measured for each plot (9 subsamples per plot). From each subsample, intact and representative leaf samples including the smaller, youngest shoot (3–14) were used to measure leaf length, leaf width, leaf area and leaf area index (LAI). The leaves were either scanned (Canon Lide 110 digital scanner) or photographed. Leaf descriptors were measured with ImageJ software (version 1.53a; Abramoff et al., 2004).

### Plant Collection for Biochemical Parameters

Several ramets were randomly collected at different locations within each plot. AG and BG tissues were separated and immediately frozen at −20°C until further analysis for AG and BG nutrient content, stable carbon and nitrogen isotope analyses (δ^13^C and δ^15^N), as well as total non-structural carbohydrate (NSC) reserves such as starch and sugars.

### Carbon and Nitrogen Tissue Content and Natural Stable Isotopes

Before analysis, the leaf and rhizome samples were freeze-dried for 48 h at the ZMT’s facilities. The dried leaf and rhizome tissues were ground and total C and N content was measured using a Euro EA 3000 elemental analyzer (EuroVector) with Acetanilid 5 (Hexatech) used as standard. Measurement accuracy was ensured by repeatedly measuring standards with known C and N concentrations (Low Soil Standard OAS 5; IVA). Carbon and nitrogen contents were expressed as a percentage of dry weight and the values were used to calculate the *C*:*N* ratios. Percent tissue phosphorus was analyzed using the wet alkaline persulphate digestion technique method on a TECAN M200Pro plate reader after Hansen and Koroleff (2009).

Stable isotopic ratios of carbon (δ^13^C/δ^ 12^C) and nitrogen (δ^15^N/δ^14^N) in samples were analyzed using a Finnigan Delta Plus mass spectrometer coupled with a Flash EA 1112 elemental analyzer. Results of isotopic composition in samples were expressed as following:

(1) δX(‰)=[(R/sampleR)reference-1]×1000,

where *X* is either δ^15^N or δ^13^C, and R is the ratio of δ^15^N/δ^14^N for nitrogen and δ^13^C/δ^12^C for carbon. Reference materials IAEA N1 and N2 (nitrogen) and USGS 24 and NBS22 (carbon) from the International Atomic Energy Agency were used for calibration. The precision of the measurements was <0.06‰ for both C and N isotopes. All δ^13^C and δ^15^N values were normalized to the international standards of wheat flour (carbon [δ^13^C]: −27.21‰; nitrogen [δ^15^N]: 2.85‰) and high organic sediment (carbon [δ^13^C]: −26.07‰; nitrogen [δ^15^N]: 4.4‰).

### Carbohydrate Reserves

Triplicate subsamples of the rhizomes were pooled and freeze-dried for 72 h. Subsequently, the samples were ground with mortar and pestle. The dried powder (0.02–0.03 g) was suspended in 1.5 mL Milli-Q water and soluble sugars were extracted from the ground dry tissues by vortexing and shaking for 15 min. Samples were centrifuged (13,000 rpm, 5 min) and the supernatant was used for soluble sugar determination via the Anthrone Assay (Viles and Silverman, 1949). Starch contents (total non-structural carbohydrates) in the remaining pellets were boiled in Milli-Q (10 min at 100°C) to gelatinize the starch. Subsequently, samples were hydrolyzed by the enzyme alpha-amylase (80 min at 80°C). The supernatant containing oligosaccharides and/or glucose broken down by the enzyme was taken and by boiling the sample material under acidic conditions (addition of 96% H_2_SO_4_) for 1.5 h at 100°C, the remaining polysaccharides were broken down to glucose molecules. Starch and sugar contents in the extracts were determined spectrophotometrically (620 nm) using an anthrone-sulphuric acid assay with a F200-Pro TECAN plate reader. Carbohydrate concentrations were quantified as sucrose equivalents (Viana et al., 2020) using sucrose calibration curves (Standard sucrose 99%, from Sigma-Aldrich). Samples of cellulose, glucose and starch were used as reference samples.

### Sediment Natural Stable Isotope Composition

From each plot of the four sites, surface layer sediments were collected in July and December 2019 in polystyrene Biological Specimen Containers. After collection, samples were stored at −20°C until further analysis.

Upon arrival at the ZMT, samples were dried at 60°C for 48 h and dry sediments were subsequently ground with a ball mill. Ground samples were then acidified with 37% HCl vapor to remove carbonates and stable isotopic composition measured as described under Section 2.7.

### Statistics

Statistical analyses were performed in R (R Core Team, 2019). For the statistical analysis, we combined data for Site 1 and 2 (SB sites) together as low impacted condition and Site 3 and 4 (NB sites) together as high impacted condition. Although, there were site- specific differences, we aimed to focus on large- scale patterns and therefore only distinguished between the environmental history of the two locations (i.e., SB vs. NB). Prior to analysis, we subset our data to values that were collected in July 2019 (start of the experiment) and December 2019 (end of the experiment). Since the *in situ* fertilization treatment started in July, it was assumed there was no nutrient treatment effect in July. We used two linear mixed effects models (LMM). One to determine if there were seasonal effects over the time of the experiment (1). For this analysis, we only looked for changes in the control plots over time and added site as a random effect. The second linear mixed effects model was performed to look at the differences between the conditions of the sites (high [NB] and low [SB] impacted sites) in July (2) and December (3), the effect of treatment- differences between fertilized and control plots (4) as well as their possible interactions (5). We performed both linear mixed effects models using the lme4 function in R (Bates et al., 2015). As fixed effects, we considered condition (high or low impacted), treatment (fertilization and control) and the interaction of condition and treatment. We added site as a random effect to our linear mixed effects models to account for the variability in sites within areas of different conditions (high or low impacted). Differences in shoot densities and leaf numbers among the sites were determined with a generalized linear mixed-effects model (GLMM), specifically a negative binomial model (link = log) because the data were counts and found to be over-dispersed (Zuur et al., 2009). The GLMM was fitted using the MASS package (Venables and Ripley, 2002). The results of the models were considered to be significant with a p value < 0.05. All models were validated visually with plots of model residuals [fitted values vs. absolute residuals (homogeneity of variance), a quantile–quantile (q–q) plot (i.e., probability plot), comparing the distribution of the standardized residuals to the normal distribution (normality), and a lag plot of the raw residuals vs. the previous residual (independence; Zuur et al., 2009)]. We assessed the significance of each independent variable (or interaction) in each model by using the likelihood ratio (LR) test that compared models with our variables of interest to the null or reduced model (Winter, 2013). To further support the results obtained by the LR test, we identified the models that best predicted the changes in seagrass indicators by minimum Akaike Information Criterium with correction for small sample sizes (AICc), model ranking and weighing (Burnham et al., 2011; Symonds and Moussalli, 2011). Models were considered the superior model if they had the lowest AICc units (determining the model strength) as well as the highest Akaike weight (AICcWt) which defines the weight of evidence of each model relative to the null or reduced model (Arnold, 2010).

Environmental parameters were analyzed separately for July and December 2019 with the ANOVA aov() or Kruskal–Wallis function in R, and a subsequent paired *t*-test with holms correction (*post hoc* test) was performed to look for significant differences between low (S1 + S2) and high (S3 + S4) impacted sites.

## Results

### Environmental Variables

**FIGURE 3:**
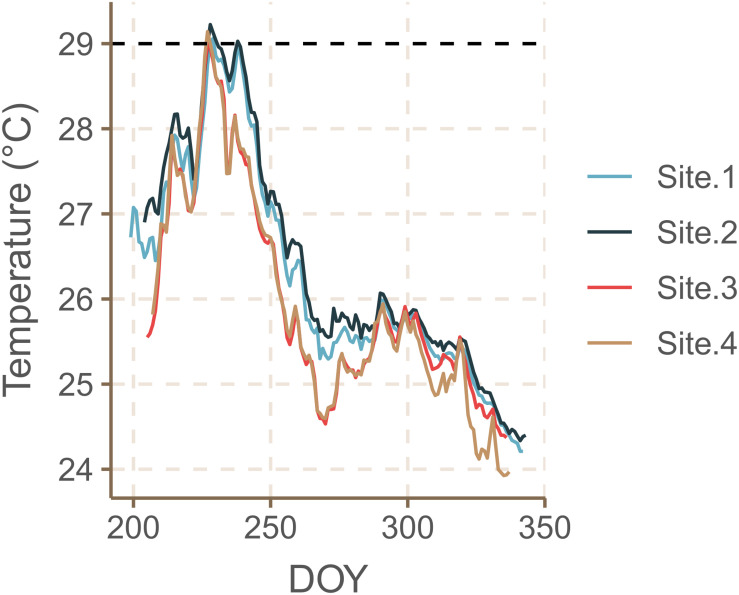
Daily average water temperatures measured at the South Beach (sites 1 and 2) and North Beach (sites 3 and 4) sites at approximately 10 m water depth recorded from 18.07–09.12.2019 (DOY = Day of the Year). The dashed line at 29°C indicates the physiological threshold, beyond which *H. stipulacea* plants were found to reduce growth rates, leaf sizes and the loss of shoots (Nguyen et al., 2020; Viana et al., 2020).

Salinity ranged between 40.4 and 40.6 psu and pH ranged between 8.22 and 8.23 with no significant differences between sites (Table 1). Daily average water temperatures reached its maximum on August 15th–16th 2019 for SB sites at 29.14 ± 0.03°C SE and for NB sites at 29.07 ± 0.47°C SE at approximately 10 m water depth (Figure 3). Temperatures over 29°C persisted for 15 consecutive days in SB Site 1, for 30 days in SB Site 2, for 8 days in NB Site 3 and for 6 days at NB Site 4. From mid-August onward, temperatures began to gradually decrease, reaching their minimum values of approximately 24°C in December 2019.

**TABLE 1:** Environmental quality parameters in the seawater.

Variable	n	July 2019				December 2019			
		S1 SB	S2 SB	S3 NB	S4 NB	S1 SB	S2 SB	S3 NB	S4 NB

**Seawater**									
Chl*a*	4	0.08 ± 0.0	0.13 ± 0.0	0.17 ± 0.0✱	0.36 ± 0.1✱	n.a	n.a	n.a	n.a
SPM	4	32.7 ± 0.6	33.7 ± 0.4	37.6 ± 4.3✱	35.8 ± 0.2✱	32.3 ± 0.5	32.0 ± 0.4	32.6 ± 0.1✱	34.2 ± 1.0✱
Corg	4	46.1 ± 2.6	42.9 ± 2.0	46.0 ± 6.6	58.0 ± 6.0	107.2 ± 5.4	116.0 ± 4.4	130.7 ± 11.4	117.2 ± 6. 5
C	4	211.2 ± 39.2	167.6 ± 35.1	187.3 ± 17.5	393.1 ± 142.0	124.5 ± 7.0	133.4 ± 9.6	177.6 ± 25.0	119.7 ± 6.0
N	4	26.7 ± 5.0	20.3 ± 4.1	22.7 ± 2.4	44.5 ± 15.9	13.7 ± 0.8	12.2 ± 1.0	22.0 ± 2.7✱	16.0 ± 0.6✱
*C*:*N*	4	24.2 ± 2.3	24.9 ± 1.6	24.9 ± 1.1	26.3 ± 0.9	9.1 ± 0.3	11.3 ± 1.8	8.1 ± 0.2✱	7.5 ± 0.4✱
Salinity	1	40.5	40.6	40.4	40.5	40.5	40.6	40.4	40.4
pH	1	8.230	8.224	8.220	8.214	8.230	8.220	8.220	8.220

*Water parameters from the GF/F filter samples (Chla, SPM, Corg, C and N) are reported in μg l^–1^. Salinity is reported in psu. ✱ indicates significant differences between impacted (NB) and non-impacted (SB) sites.*

In July 2019, there were no significant differences in N and C content of water column particulates. However, both chl*a* and SPM concentrations were higher in the NB sites (Table 1 and Supplementary Table 1). In December, NB sites had significantly higher particulate N content in the water column and lower C:N than SB sites (Table 1 and Supplementary Table 2). Moreover, both NB sites had significantly higher concentrations of SPM in the water column (Table 1 and Supplementary Table 2).

There were no significant differences in nutrient concentrations in the water column and the porewater between SB and NB sites (Table 2). However, fertilized plots in the SB sites were significantly enriched in porewater P (β = 1.19; *SE* = 0.51; df = 16; *t* = 2.32; *p* = 0.0338) and N compounds (NOx; β = 0.38; *SE* = 0.22; df = 19; *t* = 1.744; *p* = 0.0973) as well as P in the water column (β = 0.02; *SE* = 0.01; df = 19; *t* = 2.44; *p* = 0.0249), confirming that our fertilization was successful.

**TABLE 2:** Inorganic nutrient concentrations in the seawater and porewater in control and fertilized plots, expressed in μmol l^–1^.

Nutrients	Site	July 2019	December 2019	
			Control	Fertilized

SEAWATER				
DIN	North Beach	1.34 ± 0.08	0.17 ± 0.06	0.35 ± 0.10
	South Beach	0.86 ± 0.06	0.55 ± 0.14	0.92 ± 0.30
NH_4_^+^	North Beach	0.89 ± 0.28	0.17 ± 0.06	0.27 ± 0.07
	South Beach	0.69 ± 0.05	0.37 ± 0.11	0.52 ± 0.22
NO_X_	North Beach	0.45 ± 0.05	0.10 ± 0.07	0.08 ± 0.06
	South Beach	0.17 ± 0.03	0.13 ± 0.09	0.53 ± 0.22
NO_2_	North Beach	0.17 ± 0.03	0.01 ± 0.02	0.01 ± 0.02
	South Beach	0.11 ± 0.03	0.03 ± 0.02	0.03 ± 0.02
PO_4_^3–^	North Beach	0.19 ± 0.03	0.22 ± 0.03	0.21 ± 0.02
	South Beach	0.15 ± 0.02	0.19 ± 0.02	0.23 ± 0.06✱
Si	North Beach	n.a	0.47 ± 0.10	0.35 ± 0.11
	South Beach	n.a	0.00 ± 0.00	0.00 ± 0.00

**POREWATER**				

DIN	North Beach	6.00 ± 0.13	1.95 ± 0.26	5.38 ± 0.49
	South Beach	7.50 ± 0.18	3.68 ± 0.37	5.18 ± 0.44
NH_4_^+^	North Beach	5.69 ± 0.13	1.48 ± 0.26	4.72 ± 0.49
	South Beach	6.89 ± 0.17	6.53 ± 0.54	4.28 ± 0.49
NO_X_	North Beach	0.30 ± 0.03	0.47 ± 0.10	0.67 ± 0.09
	South Beach	0.60 ± 0.05	0.73 ± 0.16	1.28 ± 0.14✱
NO_2_	North Beach	0.40 ± 0.04	0.05 ± 0.03	0.07 ± 0.02
	South Beach	0.65 ± 0.05	0.08 ± 0.05	0.15 ± 0.06
PO_4_^3–^	North Beach	1.52 ± 0.09	0.56 ± 0.09	0.56 ± 0.09
	South Beach	2.35 ± 0.09	1.15 ± 0.12	2.35 ± 0.32✱
Si	North Beach	n.a	26.57 ± 0.71	26.12 ± 0.66
	South Beach	n.a	13.35 ± 0.47	14.58 ± 0.34

*✱ indicates significant differences between impacted (NB) and non-impacted (SB) sites.*

### Sediment Natural Stable Isotope Composition

There were no significant differences in δ^13^C isotopic signatures of sediment contents between low (SB) and high (NB) impacted sites in July and December 2019 (Table 3). However, δ^15^N isotopic signatures of sediment contents were lower in the SB sites in both, July and December 2019 (Supplementary Table 5). δ^15^N isotopic signatures of Site 4 (NB) were even two to three times higher than the SB sites (Supplementary Table 4).

**TABLE 3:** Mean (±SE) values of sediment isotope composition from the anthropogenically impacted sites (North Beach) and low impacted sites (South Beach) in control (*n* = 6) and fertilized (*n* = 6) plots.

Isotopic composition	Site	July 2019	December 2019	
			Control	Fertilized
δ15N	North Beach	3.32 ± 0.35	3.43 ± 0.45	3.51 ± 0.49
	South Beach	1.85 ± 0.30✱	1.08 ± 0.36✱	0.83 ± 0.46✱
δ13C	North Beach	−16.00 ± 1.00	−15.12 ± 0.26**^**†**^**	−15.03 ± 0.15
	South Beach	−14.77 ± 0.66	−14.45 ± 0.12 **^**†**^**	−15.21 ± 0.47✱✢

*δ^13^C and δ^15^N are expressed as parts per thousands. ✱ indicates significant differences between impacted (NB) and non-impacted (SB) sites. **^**†**^** indicates significant seasonal differences between July and December, and ✢ indicates significant differences between control and fertilized plots.*

Sediment δ^13^C isotopic contents increased significantly from July to December (Supplementary Table 5). Fertilization had no effects on δ^13^C and δ^15^N isotopic signatures in sediments.

### Changes Between Seasons (July 2019 and December 2019) and With Fertilization

#### Percentage Cover, Shoot Density, and Biomass

**FIGURE 4:**
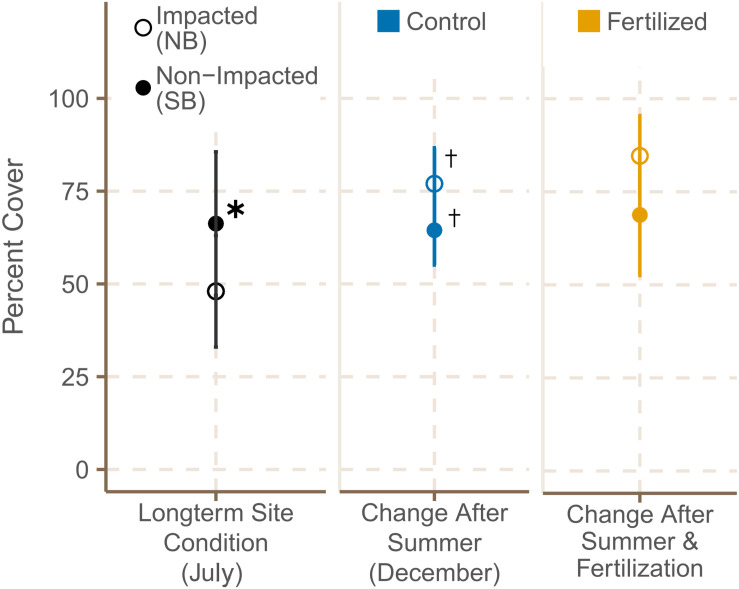
Meadow cover (%) (mean ± SE) of fertilized and control plots in July 2019 and December 2019 and * shows significant differences between impacted (NB) and non-impacted (SB) sites. **^**†**^** indicates if there were significant seasonal differences between July and December.

**FIGURE 5:**
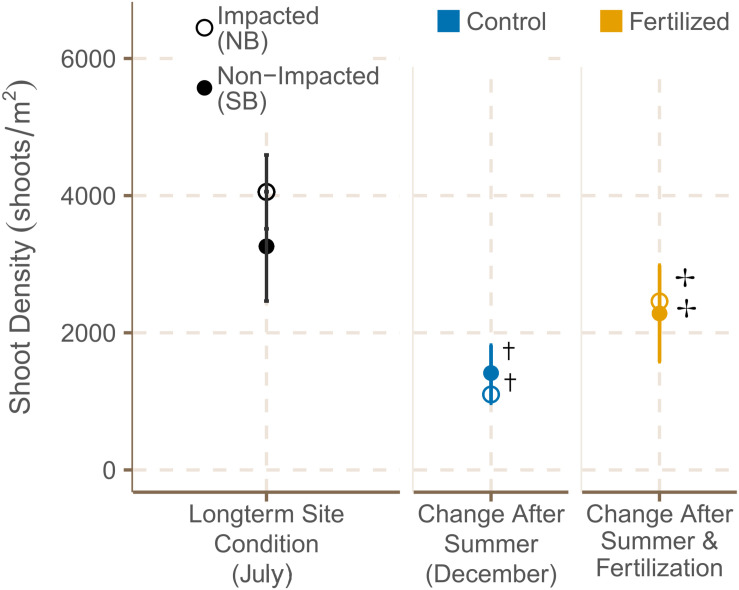
Shoot densities [shoots m^2^] (mean ± SE) of fertilized and control plots in July 2019 and December 2019. **^**†**^** indicates if there were significant seasonal differences between July and December and ✢ indicates if there were significant differences between control and fertilized plots.

Seagrass cover was higher at the SB meadows in July 2019, but increased significantly from July to December at both locations (Figure 4 and Supplementary Tables 6A, 7). Shoot density did not differ significantly between meadows in the South and North Beach in July 2019 (Supplementary Table 3). However, shoot densities showed a trend of being higher in NB sites in both July and December. From July to December 2019, shoot density was declining within both locations in control plots (Supplementary Table 7), but increased significantly in plots that had been fertilized (Figure 5 and Supplementary Table 6B).

**FIGURE 6:**
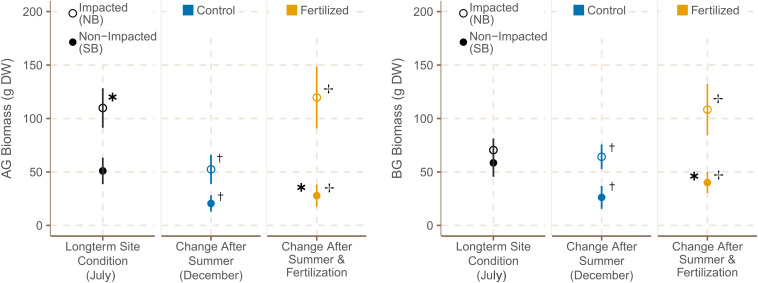
Average below and above-ground biomasses (±SE) from the *H. stipulacea* biomass cores (*n* = 12) of control (*n* = 6) and fertilized plots (*n* = 6) in July 2019 and December 2019. ✱ indicates significant differences between impacted (NB) and non-impacted (SB) sites. **^**†**^** indicates significant seasonal differences between July and December, and ✢ indicates significant differences between control and fertilized plots.

Aboveground (AG) biomass was significantly higher in NB meadows in July 2019 (Supplementary Table 7C). However, both the AG and belowground (BG) biomasses decreased significantly over the course of the experiment at NB and SB sites (Figure 6 and Supplementary Table 7). By December 2019, AG and BG biomass were both significantly higher at NB sites (Supplementary Tables 6C,D). Moreover, fertilization led to a significant increase in AG and BG biomass with a more pronounced increase at NB sites. The AG:BG ratio was lower in SB meadows in July 2019, but did not change over the course of the experiment or with fertilization (Supplementary Table 6E).

#### Morphological Parameters

Low impacted sites (SB) had a higher percentage of apical shoots in their shoot population in July 2019 (Table 4 and Supplementary Table 8D). The number of leaves per m^2^ was not significantly different in seagrass meadows from SB and NB sites in July 2019, but decreased significantly in both locations by December 2019 (Table 4 and Supplementary Table 9). However, fertilization resulted in a significant increase in the number of leaves independent of their location, but had no significant effects on percentage of apical shoots (Table 4 and Supplementary Tables 8A,D).

**TABLE 4:** Mean (±SE) values of morphological traits from *H. stipulacea* biomass cores (*n* = 12) in the anthropogenically-impacted sites (North Beach) and low impacted sites (South Beach) in control (*n* = 6) and fertilized (*n* = 6) plots.

Morphological traits	Site	July 2019	December 2019	
			Control	Fertilized
Number of leaves per m^2^	North Beach	9180.79 ± 869.42	2716.21 ± 311.12**^†^**	5595.39 ± 1010.49✢
	South Beach	8571.04 ± 1542.55	3558.24 ± 1025.06**^†^**	5622.56 ± 1628.50✢
% apical shoots	North Beach	13.30 ± 0.85	23.72 ± 1.72	15.10 ± 2.39
	South Beach	28.23 ± 2.01✱	22.55 ± 5.63	25.24 ± 3.46
Leaf length	North Beach	4.32 ± 0.05	4.12 ± 0.13	4.67 ± 0.08✢
	South Beach	2.80 ± 0.06✱	2.86 ± 0.10✱	2.86 ± 0.07✱✢
Leaf width	North Beach	1.06 ± 0.02	1.09 ± 0.04	1.25 ± 0.03✢
	South Beach	0.73 ± 0.02✱	0.71 ± 0.03✱	0.63 ± 0.03✱✢
Leaf area	North Beach	2.92 ± 0.06	2.82 ± 0.15	3.45 ± 0.10✢
	South Beach	1.19 ± 0.05✱	1.28 ± 0.07✱	2.32 ± 0.32✢
LAI	North Beach	3.29 ± 0.06	3.17 ± 0.17	3.80 ± 0.13✢
	South Beach	1.34 ± 0.02✱	1.44 ± 0.08✱	2.60 ± 0.36✢
Internodal distance	North Beach	10.44 ± 0.14	9.61 ± 1.18**^†^**	9.52 ± 0.28✢
	South Beach	8.44 ± 0.19✱	9.40 ± 0.35✱**^†^**	9.05 ± 0.37✱✢
% reproductive shoots	South Beach- S1	41.73 ± 9.93	0.00 ± 0.00	0.00 ± 0.00
	South Beach- S2	8.31 ± 2.34	0.00 ± 0.00	0.00 ± 0.00
	North Beach- S3	7.74 ± 5.99	0.00 ± 0.00	0.00 ± 0.00
	North Beach- S4	6.13 ± 1.34	0.00 ± 0.00	0.00 ± 0.00

*Leaf length, leaf width, and leaf area per shoot are expressed in cm and internodal distance in mm, while LAI is expressed as m^2^ leaf area per shoot × shoot density per m^–2^. ✱ indicates significant differences between impacted (NB) and non-impacted (SB) sites.**^**†**^** indicates significant seasonal differences between July and December, and ✢ indicates significant differences between control and fertilized plots.*

Seagrass plants growing in meadows at SB had significantly smaller and thinner leaves as well as a smaller leaf area and LAI than plants growing in NB in July and in December 2019 (Supplementary Table 9). The additional nutrient input resulted in even smaller and thinner leaves in plots at the SB sites, whereas fertilization had a positive impact on leaf length and width of plants in plots at the high impacted NB sites (Supplementary Tables 8B,C).

Seagrasses from the SB sites had significantly smaller internodal distances at the beginning and at the end of the experiment (Supplementary Table 8F). Over the course of the experiment, internodal distances were significantly reduced in all plants from both locations (Supplementary Table 9). Internodal distances of seagrasses from SB decreased even further due to fertilization. This is contrary to plants from the NB, where internodal distances increased in response to *in situ* fertilization treatment (Table 4 and Supplementary Table 8F).

Reproductive shoots were only observed in July 2019 with higher percentages in the SB Site 1 (Table 4). Male reproductive shoots (0.01% of 41.73% reproductive shoots) were only found in SB Site 1.

#### Carbon, Nitrogen, and Phosphorous Content and δ^13^C and δ^15^N Stable Isotopes

There were no differences in leaf C, N, P, and *C*:*N* of *H. stipulacea* plants between the SB and NB meadows in July and later in December 2019 from control plots (Table 5). Leaf C content and leaf *C*:*N* decreased significantly over the time of the experiment in both locations (Supplementary Tables 10A,B).

**TABLE 5:** Mean (±SE) values of biochemical traits from *H. stipulacea* shoots from the anthropogenically impacted sites (NB) and low impacted sites (SB) in control (*n* = 6) and fertilized (*n* = 6) plots.

Biochemical traits	Site	July 2019	December 2019	
			Control	Fertilized
Leaf C	North Beach	23.60 ± 0.58	20.35 ± 0.30**^†^**	20.47 ± 0.41
	South Beach	24.76 ± 0.41	21.18 ± 2.47**^†^**	23.95 ± 2.07
Rhizome C	North Beach	27.15 ± 0.28	24.81 ± 0.49	24.37 ± 0.94
	South Beach	26.04 ± 0.72	26.36 ± 2.75✱	20.69 ± 0.40
Leaf N	North Beach	1.03 ± 0.02	1.02 ± 0.03	1.19 ± 0.04
	South Beach	1.04 ± 0.04	1.10 ± 0.22	1.27 ± 0.18
Rhizome N	North Beach	0.53 ± 0.03	0.95 ± 0.12**^†^**	1.31 ± 0.17
	South Beach	0.36 ± 0.02✱	0.93 ± 0.50**^†^**	0.64 ± 0.08
Leaf δ^13^C	North Beach	−6.80 ± 0.12	−7.88 ± 0.30**^†^**	−8.07 ± 0.29
	South Beach	−6.85 ± 0.13✱	−8.22 ± 0.99**^†^**	−9.57 ± 1.16
Rhizome δ^13^C	North Beach	−7.40 ± 0.13	−7.73 ± 0.28**^†^**	−8.20 ± 0.35
	South Beach	−7.34 ± 0.16	−8.51 ± 0.96**^†^**	−7.66 ± 0.13
Leaf δ^15^N	North Beach	2.09 ± 0.26	1.16 ± 0.38**^†^**	−2.38 ± 0.40✢
	South Beach	1.37 ± 0.18	0.33 ± 0.93**^†^**	−0.52 ± 1.77✢
Rhizome δ^15^N	North Beach	1.28 ± 0.24	0.58 ± 0.38	−4.15 ± 0.46✢
	South Beach	0.75 ± 0.16✱	0.68 ± 1.15	−4.62 ± 0.50✢
Leaf *C*:*N* ratio	North Beach	22.99 ± 0.52	20.02 ± 0.70**^†^**	17.25 ± 0.57
	South Beach	24.17 ± 0.65	20.50 ± 1.62**^†^**	19.50 ± 1.07
Rhizome *C*:*N* ratio	North Beach	52.59 ± 2.88	28.69 ± 4.42**^†^**	20.63 ± 3.30✢
	South Beach	74.76 ± 3.57✱	49.54 ± 8.72✱**^†^**	35.07 ± 4.30✱✢
Leaf P	North Beach	1499.22 ± 133.35	1515.46 ± 162.22	1580.18 ± 236.69
	South Beach	1171.68 ± 132.54	1310.87 ± 191.34	1244.18 ± 249.36
Rhizome P	North Beach	1704.03 ± 50.10	1854.39 ± 208.95	1837.99 ± 198.91
	South Beach	986.41 ± 69.35✱	793.81 ± 131.69✱	802.34 ± 197.99✱
Leaf starch	North Beach	56.46 ± 3.74	44.31 ± 4.10	40.99 ± 2.99
	South Beach	88.47 ± 6.20✱	82.75 ± 23.34✱	62.08 ± 3.32✱
Leaf sucrose	North Beach	25.00 ± 1.78	22.80 ± 1.49	23.00 ± 1.76
	South Beach	52.28 ± 5.29✱	37.67 ± 4.23✱	34.00 ± 3.69✱
Rhizome starch	North Beach	74.46 ± 4.79	44.41 ± 4.71	49.11 ± 4.61
	South Beach	80.31 ± 4.01	83.05 ± 14.71✱	68.71 ± 4.41✱
Rhizome sucrose	North Beach	121.00 ± 8.31	88.15 ± 18.97	55.00 ± 14.20✢
	South Beach	113.88 ± 8.01	109.77 ± 26.41	72.00 ± 17.58✢

*C and N contents are expressed as percentages, while δ^13^C and δ^15^N are in parts per thousands. P content is shown in μg g^–1^ and starch as well as sugar contents are expressed as sucrose equivalent mg per g dry weight. ✱ indicates significant differences between impacted (NB) and non-impacted (SB) sites.**^**†**^** indicates significant seasonal differences between July and December, and ✢ indicates significant differences between control and fertilized plots.*

Rhizome tissue of seagrasses in the low impacted SB sites had significantly lower nitrogen content and higher *C*:*N* ratios in July 2019 (Supplementary Tables 10E,G). Moreover, plants from the SB had lower P contents in their rhizomes in the beginning and in the end of the experiment (Supplementary Table 10H).

Rhizome N content increased significantly over the course of the experiment and as a response the *C*:*N* of rhizomes decreased significantly in all sites (Supplementary Table 11). Plants from the low impacted sites had still significantly higher rhizome *C*:*N* as well as higher C contents in December than their *H. stipulacea* counterparts from the NB site (Supplementary Tables 10E,F). Fertilization resulted only in significantly lower *C*:*N* in rhizome tissues of seagrasses from both locations (Supplementary Table 10E).

Plants from SB meadows had significantly lower δ^13^C isotopic signatures in leaves and significantly lower δ^15^N isotopic signatures in rhizomes than seagrasses from the NB sites in July 2019 (Table 5 and Supplementary Tables 12A,D). There were no significant differences in δ^13^C and δ^15^N isotopic contents in both leaf and rhizome tissues between plants growing in the SB and NB sites in December 2019 (Table 5). However, leaf and rhizome δ^13^C as well as leaf δ^15^N isotopic contents significantly decreased from July to December within both sites (Supplementary Table 12E). Fertilization resulted in a significant decrease of δ^15^N isotopic signatures in both leaf and rhizome tissues in SB and NB sites.

#### Non-structural Carbohydrates

The main form of NSC was sucrose in the rhizome tissues, whereas starch was the more dominant NSC in the leaf tissues.

##### Leaves

**FIGURE 7:**
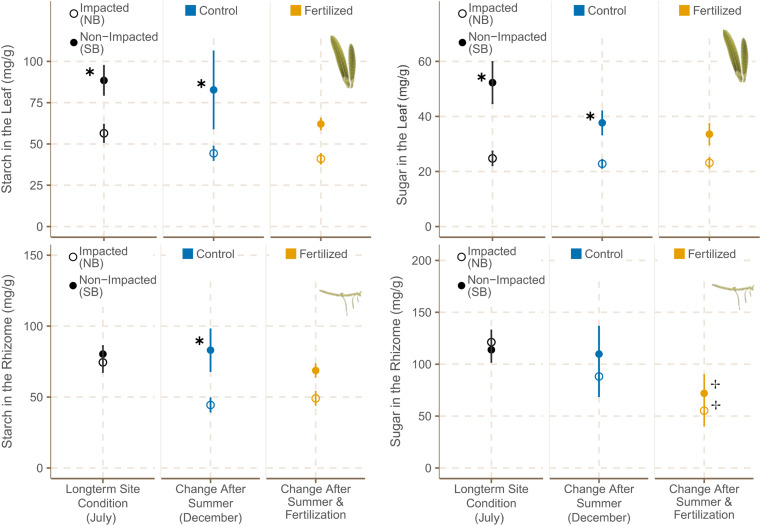
Starch and sugar concentrations [sucrose eq mg g (DW)^– 1^] in leaves (mean ± SE) and rhizomes (mean ± SE) of *H. stipulacea* in fertilized (*n* = 6) and control plots (*n* = 6) from different sites in July and December 2019. ✱ shows significant differences between impacted (NB) and non-impacted (SB) areas and ✢ states if there are differences between control and fertilized plots.

Plants growing in the SB sites had significantly higher starch and sugar contents in their leaf tissues at the beginning and at the end of the experiment (Figure 7 and Supplementary Tables 13A,B).

##### Rhizomes

Sugar and starch contents did not differ between seagrass meadows from NB and SB sites at the start of the experiment. However, sugar was the dominant form of NSC in rhizome tissues of NB plants whereas SB plants had higher starch contents in their rhizomes tissue by December. Starch content in rhizome tissues of seagrasses growing in the SB sites was significantly higher at the end of the experiment than the one of plants growing in the NB (Figure 7 and Supplementary Table 13D). Fertilization resulted in a significant reduction of sugar contents in the rhizomes of seagrasses from both locations (Table 5 and Supplementary Table 13C). Moreover, starch concentrations in the rhizomes of SB plants also showed a decreasing trend with fertilization (Supplementary Table 4).

### Principal Component Analysis (PCA)

**FIGURE 8:**
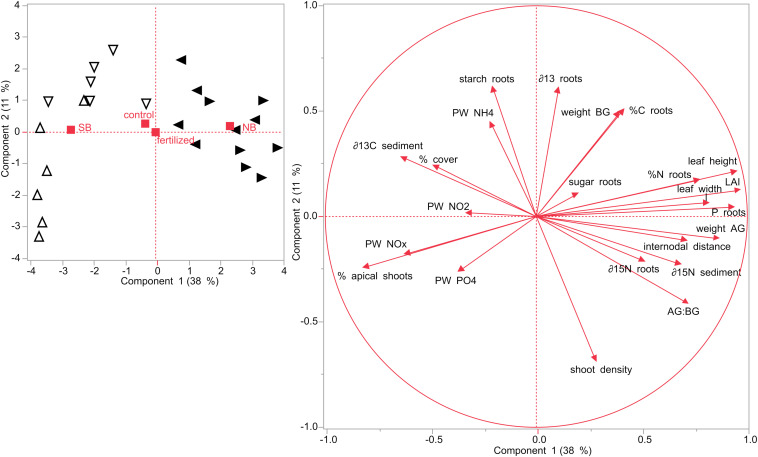
Principal component analysis (PCA) performed on seagrasses from impacted (NB) and non-impacted (SB) sites at the start of the experiment in July 2019. Different symbols refer to the different sites: Δ site 1 (SB), ∇ site 2 (SB), ◀ site 3 (NB) and ▶ site 4 (NB).

Principal component analysis were performed for the two different sampling months, July and December. In July, the first two components of the PCA explained roughly 50% of the variability (Figure 8). Seagrass plots that were assigned to the two treatments (fertilized and control) did initially not differ from each other at the start of the experiment in July 2019. However, there was a distinct difference between the two locations, NB and SB. Also, plots from each of the four sites clustered together, but the two sites within one location (S1 + S2 and S3 + S4) slightly overlapped, showing that they are similar to each other. SB sites were characterized by higher porewater nutrient concentrations, percent cover, starch content in their rhizomes and roots and higher δ^13^C isotopic contents in their sediments. NB sites were characterized by larger leaf length, width, LAI and internodal distances as well as increased nutrient content (%N, %C, and P content) and sugar concentrations in their rhizomes and roots. Moreover, plants in the NB sites were related with increased AG and BG biomass, higher AG:BG ratios and were enriched in δ^15^N isotopic contents in their rhizomes as well as in their sediments.

**FIGURE 9:**
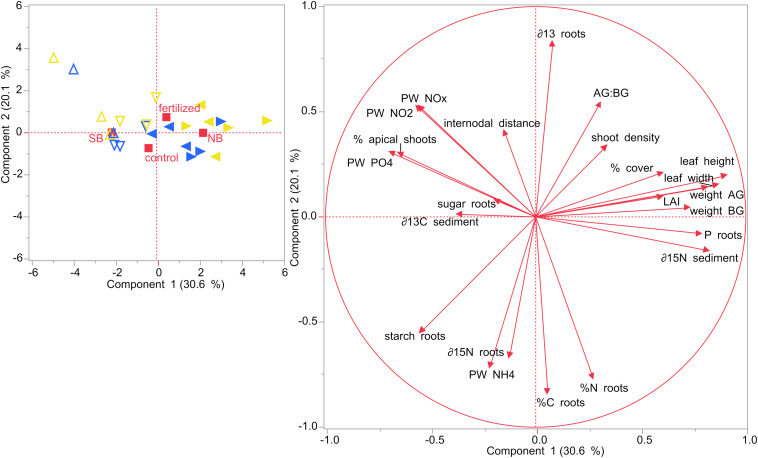
PCA performed on seagrasses from impacted (NB) and non-impacted (SB) sites at the end of the experiment in December 2019. Different symbols refer to the different sites: Δ site 1 (SB), ∇ site 2 (SB), ◀ site 3 (NB) and ▶ site 4 (NB). Yellow colored symbols depict fertilized plots while blue colored symbols show control plots.

In December, SB and NB sites were clearly separated as well as the control and the fertilized plots, although these latter plots showed some slight overlap (Figure 9). The first two components of the PCA explained more than 50% of the variability. Plants growing in the SB were related with higher sugar concentrations in their rhizomes and roots and higher δ^13^C isotopic contents in their sediments. SB plants from control plots were also characterized by higher starch concentrations in their rhizomes and higher δ^15^N isotopic contents in their rhizomes, while SB plants in fertilized plots were associated with a higher percentage of apical shoots in their shoot populations and higher porewater P and N compounds.

On the other hand, plants from fertilized plots in the NB sites, were positively correlated with increased AG and BG biomass and higher P content in their rhizome and roots as well as larger leaf length, width and LAI. Seagrasses from fertilized plots in the impacted NB sites were positively correlated with higher AG:BG ratios as well as increased shoot densities and percent cover. Seagrasses from control plots in both sites were positively correlated with higher rhizome and root tissue nutrient content (%C and %N).

## Discussion

Our experimental design investigating changes in morphological, biochemical, structural and population level traits proved useful for the identification of indicators for nutrient stress in the tropical seagrass *H. stipulacea* in its native range (northern GoA). It is likely that the effects of fertilization would have resulted in more significant changes in our chosen seagrass indicators if further temporal and spatial replication would have been possible. Despite these limitations, our approach was successful for highlighting the different responses to eutrophication among *H. stipulacea* meadows growing under different levels of anthropogenic pressures (Mejia et al., 2016; Winters et al., 2017), while enduring summer temperatures that were higher than normal, and which potentially surpassed the threshold for *H. stipulacea*’s optimal growth (Nguyen et al., 2020). Our findings provide a good example of the response of a fast-growing opportunistic seagrass species undergoing various levels of local anthropogenic stressors and clearly show the difference to responses of a large-bodied, slow growing seagrass species in a previous study (Helber et al., 2021).

To better compare and visualize the changes in population level metrics and individual plant morphological and biochemical traits that occurred over the season and with the fertilizer treatment, we summarized the most important indicators in Table 5. Population metrics of plants from both locations showed generally negative trends. However, the effects of fertilization were strongly dependent on the meadows’ location and/or contrasting levels of eutrophication history. Fertilization had mainly positive effects on plants from the NB, often resulting in antagonistic effects from seasonal differences, possibly due to temperature stress. In contrast, fertilization negatively affected seagrasses from SB, reinforcing the negative trends of seasonal changes or temperature stress (Table 6).

**TABLE 6:**
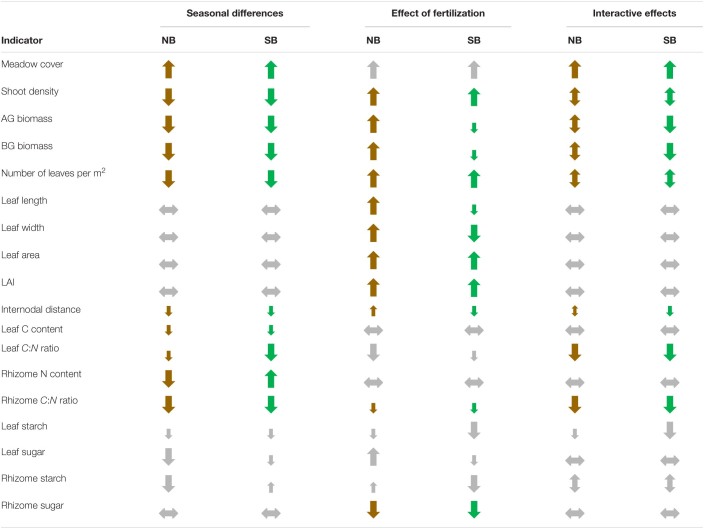
Summary of significant differences in responses of the most important indicators affected by season and by the addition of fertilizer as well as their interactive effects.

*Statistically significant effects on seagrasses from the high impacted sites in NB are represented by brown color while statistically significant effects on plants from the low impacted sites in the SB, are represented by the green colors. The direction and size of the arrows represent the direction and strength of the effects, respectively. Gray arrows represent trends that were not statistically significant (p > 0.05).*

*Halophila stipulacea* populations from the NB and SB sites showed different responses to *in situ* fertilization even though these two sites are geographically rather close (6–8 km). The *in situ* fertilization treatment, simulating chronic eutrophication had mainly negative effects on seagrass plants from the SB sites, a marine protected area, while *H. stipulacea* populations from the anthropogenically impacted NB sites benefited for the most part from the additional nutrient input (Table 6). Lower AG and BG biomasses, reduced LAI, smaller internodal distances, high sexual reproductive effort and the increasing occurrence of apical shoots in seagrasses from SB sites might indicate that the *H. stipulacea* plants are stressed or not growing under optimal conditions at the SB sites. The addition of fertilizer in the SB sites seemed to have pushed plants even further away from their optimal conditions as shown by the reductions in AG and BG biomass and internodal distances. Indeed, these differences between the two sites might suggest yet again that the SB region might not represent the ideal environment for *H. stipulacea* plants, as hypothesized by Mejia et al. (2016) and Beca-Carretero et al. (2020a). While a population genetics study has so far not been conducted in the northern GoA, genotypic selection of *H. stipulacea* plants that are able to grow within eutrophic conditions in the NB sites might have resulted in the locally adapted populations from the NB as demonstrated for other seagrasses growing in areas under high disturbances (Diaz-Almela et al., 2007; Arnaud-Haond et al., 2010; Connolly et al., 2018).

However, plants from the NB sites might experience negative effects by the reduced light penetration in the future as an indirect effect of increasing nutrient pollution. First signs of a compromise in the plants’ carbon balance could be detected by a strong decrease in their rhizome’s starch contents. Thus, we identified carbohydrate reserves in leaves and rhizomes, LAI, internodal length, reproductive effort and percentage of apical shoots as suitable indicators for stress in this seagrass species in its native range. Additionally, light penetration at sites that receive high nutrient inputs should be monitored. Even more important would be to monitor the maximal depth boundaries, which would be directly related to the local water quality (Nielsen et al., 2002).

### Environmental Conditions Across Sites

Unexpectedly, we did not find any significant differences in water and porewater nutrient concentrations between the SB and NB sites. Higher nutrients at the SB could be related to the neighboring coral reefs, which may add additional nutrients to the area (Silverman et al., 2007; Mejia et al., 2016). Water column nutrient concentrations might not have shown differences between the sites because of the prevailing current regimes. The current in the GoA moves southwards on the western coast leading to a faster dilution of nutrients in the North Beach and at the same time accumulating a higher amount of nutrients in the South Beach (Abelson et al., 1999). Moreover, biogeochemical differences in sediment characteristics (NB, muddy and dense; SB, sandy and loose) and different composition in sedimentary benthic faunal communities might have influenced nutrient dynamics at both sites (Gilad et al., 2018). While we did not find any proof of nutrient enrichment in the water samples of the fertilized NB plots, we did, however, find that fertilized plots in the SB sites were significantly enriched in porewater P and N compounds as well as P in the water column, confirming that our fertilization was successful. The fact that the δ^15^N isotopic signatures in the tissue samples decreased in fertilized plots in the NB and SB sites, further confirms that the fertilization was successful in both sites (detailed below; Table 5). Previous studies (Armitage and Fourqurean, 2009; Teichberg et al., 2013) measured lighter δ^15^N isotopic signatures in macroalgae and seagrasses in response to the addition of artificial fertilizer, which have δ^15^N isotopic signatures close to 0‰ (Fourqurean et al., 2005). These results suggest that sampling of water column and porewater nutrients would have probably required a more substantial spatial and temporal sampling in order to detect the effects of the fertilizer addition. It has been shown in previous studies that rapid dilution processes and high phytoplankton grazing resulting in a fast transfer of dissolved nutrients through the food web to higher trophic levels makes it harder to detect signs of eutrophication (Dalsgaard and Krause-Jensen, 2006; Pitta et al., 2009).

The highest temperatures were observed in August with daily average values of 27.88 ± 0.07°C SE at the NB sites and 28.33 ± 0.07°C SE at the SB sites at around 10 m water depth. *In situ* monitoring of water temperature measured at 2 m depth on the IUI’s pier confirms that August 2019 water temperatures were 1.0–2.5°C warmer than temperatures normally measured during this month over the last 11 years (the Israel National Monitoring Program at the Gulf of Eilat – Available Data, 2020).

### The Morphological and Community-Level Responses of *H. stipulacea* Populations to *in situ* Fertilization

Before we started our experiment in July 2019, there were no significant differences in shoot densities between seagrasses from SB and NB sites. However, meadow cover was higher in SB sites in the beginning of the experiment, but by December there were no differences in meadow cover between SB and NB sites. Plants from the NB sites produced more AG biomass than SB plants, most likely caused by the larger leaves of plants growing in the NB sites (2.92 ± 0.06 cm^2^) compared with the leaf sizes of plants growing in the clearer waters of the SB sites (1.19 ± 0.05 cm^2^) (Table 4; Mejia et al., 2016; Rotini et al., 2017). Seagrasses from NB populations have adapted to their low irradiance environment and high water turbidity and as a response produced longer and wider leaves resulting in a higher leaf surface area and LAI, as also reported in recent studies (Mejia et al., 2016; Azcárate-García et al., 2020; Beca-Carretero et al., 2020a). This adaptive efficacy helps to improve their carbon balance by increasing their photosynthetically active surfaces (Erftemeijer and Stapel, 1999; Ralph et al., 2007). Indeed, starch content was high in leaf tissues indicating that *H. stipulacea* and especially the NB populations store energy in the form of starch in their leaves, which they can use for new leaf growth and for enlarging their photosynthetically active surfaces.

The addition of fertilizer had different effects on seagrass meadows from low and high impacted sites. Percent cover and shoot density significantly increased in both sites as a response to the additional nutrient input compared to control plots. However, while the additional nutrient input resulted in a significantly enhanced biomass production in plants from the NB, there was only a small trend of an increase in biomass of seagrasses growing in fertilized plots in the SB sites.

### Individual Plant Responses as Indicators of Stress

#### Morphological Traits

Internodal distances of plants growing in the SB sites were smaller than the ones of seagrasses from the NB, and decreased further with fertilization, probably as a response to nutrient stress. Shorter internodal distances have been shown to be a common response to environmental stress and might serve as a proxy of increased shoot and/or root densities (Kilminster et al., 2008; Holmer et al., 2011; Viana et al., 2020). This is in accordance with seagrasses from the SB having shorter internodal distances and investing more energy in roots than in leaf formation, as shown by their reduced AG:BG ratio. Seagrasses from the SB had a higher percentage of apical shoots as well as reproductive shoots in their shoot population in July 2019. Reproductive shoots were only found in July and seagrass populations at both locations were female-biased, confirming previous studies (Malm, 2006; Nguyen et al., 2018). In their review across thirty-two studies for 11 seagrass species, Cabaço and Santos (2012) showed that an increase in sexual reproductive efforts were strongly associated with responses to stress or when plants are growing in unfavorable environments. Furthermore, the predominant presence of apical shoots might also indicate that the plants are not growing under optimal conditions at the SB sites (as discussed above). There was a trend of a further increase in apical shoots with fertilization at the SB sites, while the opposite trend was observed in seagrasses from NB (Table 4). It has been shown that plants exposed to stressors have a higher number of apical shoots in their shoot population to secure the persistence of their population (Ruocco et al., 2020). In this way, seagrasses are able to spread and find more favorable habitats (Meinesz and Lefevre, 1984; Ruocco et al., 2020).

Moreover, seagrasses from SB had smaller and thinner leaves and a smaller LAI. LAI combines individual plant traits (leaf area and number of leaves) with population level metrics (shoot density) that have been shown to decrease in response to anthropogenic pressures (Guidetti, 2001; Leoni et al., 2006; Karamfilov et al., 2019; Kletou, 2019; Zulfikar and Boer, 2020). LAI was found to be the most significant determinant of ecosystem primary production and negative changes might thus have devastating impacts on seagrass ecosystems such as impairing their carbon sequestration capacity (Asner et al., 2003; Barr et al., 2004; Saigusa et al., 2005; Wang et al., 2019).

#### Nutrient Content and Isotopic Signatures (δ^15^N and δ^13^C)

Seagrasses from both NB and SB sites seem to be N- limited in the oligotrophic conditions of the GoA, since %N in leaves and rhizomes was well below 1.8% DW as previously reported (Duarte, 1992; Cardini et al., 2018; Beca-Carretero et al., 2020a). The N as well as P contents were also significantly lower in rhizomes of plants from the two SB sites, suggesting that NB sites receive higher nutrient inputs than the protected SB sites. Additionally, seagrass tissues from NB sites were markedly enriched in δ^15^N in both their leaf and rhizome tissues (Supplementary Table 4). Seagrasses from fertilized plots in December were markedly depleted in δ^15^N in both their leaf and rhizome tissues (Supplementary Table 4), further confirming the fertilization success as synthetic fertilizers have δ^15^N signatures ranging from −2‰ and 2‰ (Bateman and Kelly, 2007). Sedimentary δ^15^N was shown to be a more reliable indicator for changes in anthropogenic pressures and the exposure to sewage effluents was reflected in δ^15^N values of 4–6‰ which were measured in sediments of Site 4 in the NB area (Supplementary Table 4; Ruiz-Fernández et al., 2002; Lapointe et al., 2004; Román et al., 2019). In contrast, plants from the two SB sites had significantly lower δ^15^N isotopic signatures in their underlying sediments. This further indicates that the NB plants might be exposed to land-based anthropogenic nutrient inputs [suggested also by Winters et al. (2017) as N content in seagrass tissue was shown to be an indicator for their long-term nutrient exposure (Udy et al., 1999; Fourqurean et al., 2006; Mejia et al., 2016; Beca-Carretero et al., 2020a)]. This area is also exposed to sewage run-off, the effects of flash floods, and the Kinnet Canal that bring nutrients into the local meadows (Mejia et al., 2016; Winters et al., 2017). In July as well as in December we found higher SPM concentrations in the water column of the NB meadows. Higher nutritional content of leaves resulted often in increased consumption rates of herbivores and leaf palatability was observed to be greater under chronic nutrient pollution and in combination with warming and acidification (Jiménez-Ramos et al., 2017; Campbell et al., 2018; Ravaglioli et al., 2018). However, mesograzers (such as small crustaceans) were shown to buffer eutrophication effects by consuming epiphytic algae that would otherwise overgrow seagrass leaves and compete with the plants for light and nutrients (Heck et al., 2006; Reynolds et al., 2014). We did not measure grazing pressure and/or quantify herbivore communities at SB and NB sites. However, grazing activity might be indeed higher at NB sites as those seagrasses had a significantly higher percentage of leaves with lost apex (Mejia et al., 2016).

The long-term exposure to nutrients in the NB area, caused not only by the proximity of the hotel strip and marina, but also by the sporadic sewage run-offs and winter flash floods (Katz et al., 2015; Winters et al., 2015), might have resulted in creating a rather nutrient “spoilt” *H. stipulacea* population, that is always in demand for even more nutrients. Plants growing in those sites seem to benefit from the additional nutrients since their N demands are not met in their environment. Our results are in agreement with previous studies in the Caribbean showing that *H. stipulacea* is able to form extremely dense mats under increased nutrient conditions even in sulfidic sediments (van Tussenbroek et al., 2016).

#### Carbohydrates

Plants from SB had higher starch concentrations in their rhizomes by December 2019. In contrast to plants from NB, fertilization seemed to negatively impact seagrasses from SB, resulting in a small decrease of SB plant’s rhizome starch content of around 14%. The considerable depletion of rhizome starch contents (reduction of 35–40%) 5 months after the summer heat peak in *H. stipulacea* populations in NB might have been the result of the constrained light penetration during the summer months on these sites as evidenced by the higher water turbidity (Supplementary Figure 1) and the higher chl *a* concentrations measured (Roca et al., 2016; Krause-Jensen et al., 2020; Supplementary Figure 1). Under low light availability, plants do not meet their energy demands through photosynthesis and need to use up their carbohydrate reserves (Alcoverro et al., 2001; Ruiz et al., 2001). Additionally, future warming (already occurring in the northern GoA; Fine et al., 2013; Nguyen et al., 2020) will likely further increase respiration rates (Ryan, 1991; Rasmusson et al., 2020), thereby increasing the light requirements of *H. stipulacea* populations in NB. This might cause a negative C budget which might lead in the future to a further depletion of their carbohydrate reserves (Lee et al., 2007; Beca-Carretero et al., 2018; Krause-Jensen et al., 2020). In addition to the remobilization of carbohydrates, seagrasses are able to cope with reduced light availability by increasing their rate of carbon fixation per unit biomass as shown in the morphological adaptations (longer and wider leaves) and by the increased photosynthetic pigment content of plants growing in NB sites (Mejia et al., 2016; O’Brien et al., 2018a). These populations receive less light than seagrasses growing in the SB; however, the light reduction at 10 m is not too limiting as the meadows reach down to 30 m depths (Winters et al., 2017) and our plots were established at around 10 m depth.

Seagrasses growing in the low impacted SB sites also experienced a depletion of their leaf sucrose content by 28–35%, which has been considered a general indicator for stress in different seagrass species (Roca et al., 2016). Recent climate change simulations based on mesocosm studies have shown that *H. stipulacea* populations from NB experienced temperature stress and reduced fitness after a 2 weeks exposure to temperatures of beyond the possible thermal threshold of 29°C, resulting in reduced growth rates, leaf sizes and the loss of shoots (Nguyen et al., 2020). Additionally, it was shown in a mesocosm experiment with *H. stipulacea* from the Indian Ocean that 31°C is above its thermal optimum (Viana et al., 2020). Maximum temperatures close to those (29.7–30.4°C) have been reached at 10 m depth over the summer months (6–30 consecutive days depending on the site) in 2019 in the northern GoA and may be also a reason for the observed decrease in their rhizome starch contents.

## Ecological Implications

Known for its ability to grow in a wide range of light conditions (i.e., depths), salinities, temperatures, and substrates [reviewed by Winters et al. (2020)], our findings show that *H. stipulacea* is also able to acclimate to environments with different levels of nutrients confirming its high plasticity (Viana et al., 2020; Winters et al., 2020). This plasticity was mostly associated with morphological and biochemical responses of *H. stipulacea* to the experienced stressors. In addition to the response to the nutrients themselves (i.e., the *in situ* fertilization treatment; discussed above), changes in structural and demographic/population-level traits occurred also as a response to different seasons. Nutrient enrichment, as shown in our study, combined with summer temperatures that surpassed the threshold for optimal growth, had only negative effects on performance of plants from the more pristine SB sites, located within a marine protected area. Seagrass plants growing in the protected SB sites showed first signs of stress under the simulated eutrophication and high summer temperatures by reduction in NSC, sugar (decline of 37%) and starch (decline of 15%) contents of their rhizomes. Furthermore, plants in SB demonstrated declining internodal distances and an increase in the frequency of their apical shoots within their shoot population, which are confirmed indicators for stress in this and other seagrass species (Kilminster et al., 2008; Holmer et al., 2011; Ruocco et al., 2020; Viana et al., 2020). Moreover, LAI of NB populations was approximately twice as high as the ones of *H. stipulacea* growing in SB at the same depth. LAI in low impacted sites decreased even further with fertilization, indicating that seagrasses from SB sites might suffer impairments in their ecosystem functions, such as their carbon sequestration capacity, under future scenarios that include eutrophication of the oligotrophic GoA. In contrast, the limited response to nutrient stress of *H. stipulacea* populations from NB suggests that these populations are well adapted to the conditions in this environment (low irradiance, high water turbidity and high nutrients). The fact that NB populations perform even better under the additional nutrient inputs further supports previous suggestions of Cardini et al. (2018) and Beca-Carretero et al. (2020a) that *H. stipulacea* living in these vast NB meadows is actually N limited.

However, future studies should look into the genotypic and genetic diversity of local meadows, as it has been shown that reduced genotypic and genetic diversity might lead to constraints in the future adaptive potential of plants, resilience to stress, and sexual reproductive efforts (Linhart and Grant, 1996; Ehlers et al., 2008; Tiffin and Ross-Ibarra, 2014). Rhizome starch contents were significantly depleted (reduction of 35–40%) after the summer heat peak in NB seagrasses, which might have been the result of the constrained light penetration during the summer months on these sites as indicated by the high water turbidity and higher chl *a* concentrations in comparison to SB sites (Roca et al., 2016; Krause-Jensen et al., 2020; Supplementary Figure 1). With the GoA being one of the region’s most popular tourist attractions, alongside it being one of its strongest economic growth engines, large coastal development projects are taking place on both sides of the Gulf^1^. These ongoing and increasing human activities so close to local *H. stipulacea* meadows may further deteriorate water quality and light penetration. This needs to be closely monitored as temperature maxima in the GoA and their duration will increase in the future (Fine et al., 2013; Nguyen et al., 2020) close to values that are not only above their optimum growth temperatures, but even beyond their upper thermal tolerance. Thus, nutrient loading from human related activities might make seagrasses at the NB sites even more sensitive to global warming in the future.

Together with recent mesocosm simulated climate change experiments (Nguyen et al., 2020), the results of our study demonstrate that global warming and increased coastal nutrient pollution might lead to vast reductions of *H. stipulacea* populations from the SB area. In contrast, seagrasses from the NB area seemed to be mainly unaffected or performed even better under the enriched conditions. These differences between a stress sensitive population vs. a stress resilient population, are similar to recent work on native (from the Red Sea) vs. invasive *H. stipulacea* populations from Greece and Cyprus (Nguyen et al., 2020; Wesselmann et al., 2020). Using a common garden simulated thermal stress experiment, Nguyen et al. (2020) showed that native *H. stipulacea* plants were negatively affected in photo-physiological and growth responses by thermal stress, while the invasive plants did not suffer and might have even benefited from it.

Recent work by Chiquillo et al. (2018) used 2bRAD genotyping (Wang et al., 2012) and found that genotypic diversity of the invasive Mediterranean *H. stipulacea* populations was lower than in the native populations in the GoA [further indicating to a genotypic selection of the invasive population as was also shown for *Zostera muelleri* in Moreton Bay, Australia (Padfield et al., 2016; Connolly et al., 2018; Molina-Montenegro et al., 2018)]. Invasive *H. stipulacea* populations in the Mediterranean were even able to adapt their thermal niche to the colder winter temperatures experienced in their new habitat (Wesselmann et al., 2020). The ability for this rapid evolutionary adaptation and to sexually reproduce in its invasive habitat (Gerakaris and Tsiamis, 2015; Nguyen et al., 2018) makes *H. stipulacea* an exceptionally strong competitor for other macrophytes.

It was shown in recent mesocosm and field experiments (Pazzaglia et al., 2020; Helber et al., 2021) that eutrophication and higher temperatures will have detrimental effects on *P. oceanica*, the dominating key foundation seagrass species in the Mediterranean. Nitrogen load thresholds for this endemic seagrass were identified to range between 0.8 and 1.1 t N km^–2^ over 6 months (Fernandes et al., 2019). Especially in areas where plants were already exposed to higher anthropogenic pressures, *P. oceanica* activated strategies to cope with the additional nutrient input that probably were energetically costly as indicated by cutbacks in their carbohydrate reserves (Pazzaglia et al., 2020; Helber et al., 2021). In contrast, *H. stipulacea* populations from NB in the current study seem to thrive under the additional nutrient supply, similar to invasive populations in the Caribbean (Willette et al., 2014; van Tussenbroek et al., 2016; Winters et al., 2020).

Thus, while future ocean warming and the rising frequency of heat waves and high cultural eutrophication will most likely lead to further losses in *P. oceanica* meadows (Coma et al., 2009; Marbà and Duarte, 2010; Jordà et al., 2012; Chefaoui et al., 2018), emerging new habitats might be available for the colonization by better adapted macrophytes, especially the invasive *H. stipulacea* (Klein and Verlaque, 2008; Montefalcone et al., 2010; Sghaier et al., 2011; Beca-Carretero et al., 2020b). Indeed, it was predicted that climate change and anthropogenic impacts might make around 85% of the Mediterranean coastline suitable for the colonization by *H. stipulacea* (Beca-Carretero et al., 2020b). The resulting regime shift in the seagrass community might not only have far reaching consequences on ecosystem functions, but also lead to massive economic losses due to the loss of ecosystem services provided by *P. oceanica* (Vassallo et al., 2013; Campagne et al., 2014; El Zrelli et al., 2020). Our results highlight the high acclimation potential of *H. stipulacea* found growing in high nutrient environments, giving it an advantage over other seagrasses less tolerant to nutrient stress. These results have important implications to management and conservation efforts, not only in shallow coastal areas where *H. stipulacea* is the native species, but also (and maybe even more) in both its historical (Mediterranean) and the new invasive (Caribbean) habitats.

## Data Availability Statement

The original contributions generated for this study are included in the article/Supplementary Material, further inquiries can be directed to the corresponding author.

## Author Contributions

SH, GW, HR, and MT conceived and designed the experiments. EB and MiS contributed in designing the experiments. MiS, MaS, and SB assisted with the fieldwork and during sampling campaigns. SH performed the field experiments, developed methodologies, and performed all the other laboratory analyses together with SB. EB did the statistical analysis. EB and MaS did the graphs and worked on the manuscript. All authors wrote and reviewed the manuscript.

## Conflict of Interest

The authors declare that the research was conducted in the absence of any commercial or financial relationships that could be construed as a potential conflict of interest.

## Publisher’s Note

All claims expressed in this article are solely those of the authors and do not necessarily represent those of their affiliated organizations, or those of the publisher, the editors and the reviewers. Any product that may be evaluated in this article, or claim that may be made by its manufacturer, is not guaranteed or endorsed by the publisher.

## Footnotes

1
www.sarayaaqaba.com

## Supplementary Material

Supplementary Figure 1 Monthly averages of sechi disk depth [m] and chlorophyll a concentration [μg l^–1^] in 2019 at South Beach and North Beach sites. The data have been obtained from Israel’s National Monitoring Programme (NMP) of the Gulf of Eilat (http://iui-eilat.ac.il/Research/NMPMeteoData.aspx; accessed 03/06/2021).

Supplementary Figure 2 Yearly averages of sechi disk depth [m] and chlorophyll a concentration [μg l^–1^] from 2010 to 2019 at South Beach and North Beach sites. The data have been obtained from Israel’s National Monitoring Programme (NMP) of the Gulf of Eilat (http://iui-eilat.ac.il/Research/NMPMeteoData.aspx; accessed 03/06/2021).
