# Supplementary figures and images for: Nutrient History Affects the Response and Resilience of the Tropical Seagrass Halophila stipulacea to Further Enrichment in Its Native Habitat
Source: Front Plant Sci. 2021 Aug 5;12:678341. doi: 10.3389/fpls.2021.678341 (PMC8374242; doi:10.3389/fpls.2021.678341)

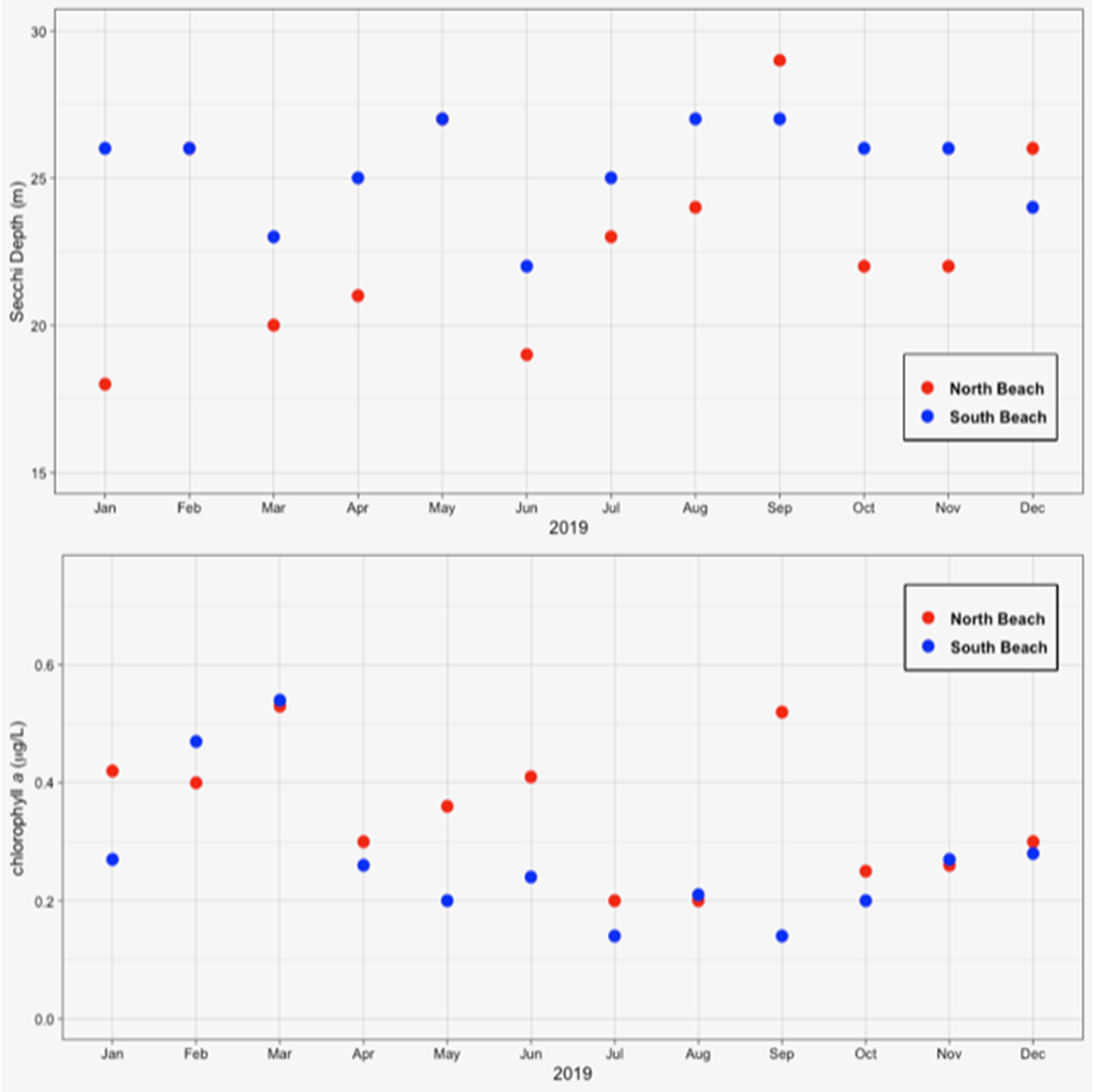

Supplement: Supplementary Figure 1 — Monthly averages of sechi disk depth [m] and chlorophyll a concentration [μg l–1] in 2019 at South Beach and North Beach sites. The data have been obtained from Israel’s National Monitoring Programme (NMP) of the Gulf of Eilat (http://iui-eilat.ac.il/Research/NMPMeteoData.aspx; accessed 03/06/2021). [file Image_1.tif]

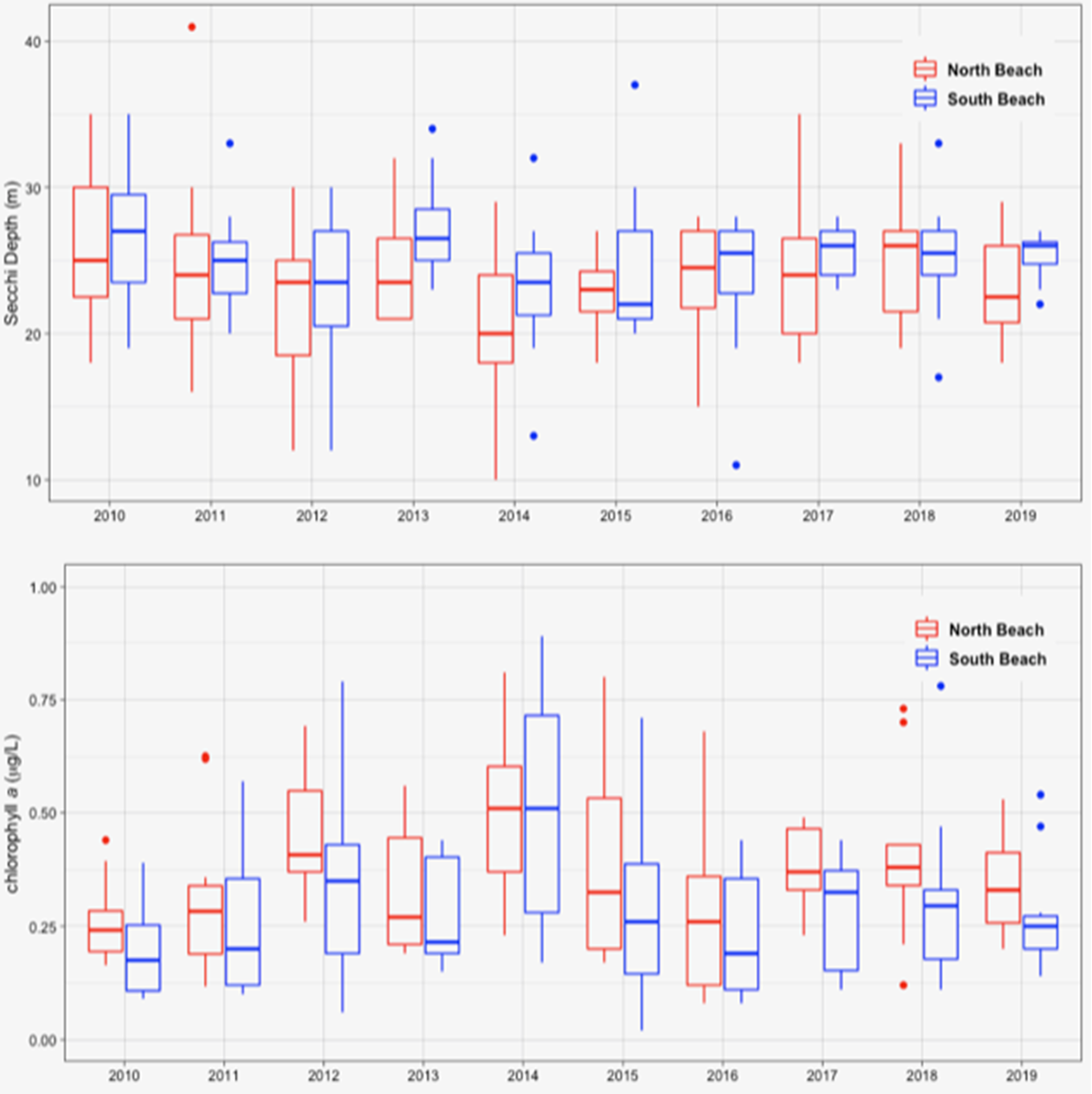

Supplement: Supplementary Figure 2 — Yearly averages of sechi disk depth [m] and chlorophyll a concentration [μg l–1] from 2010 to 2019 at South Beach and North Beach sites. The data have been obtained from Israel’s National Monitoring Programme (NMP) of the Gulf of Eilat (http://iui-eilat.ac.il/Research/NMPMeteoData.aspx; accessed 03/06/2021). [file Image_2.tif]
