# Supplementary material for: Nutrient History Affects the Response and Resilience of the Tropical Seagrass Halophila stipulacea to Further Enrichment in Its Native Habitat
Source: Front Plant Sci. 2021 Aug 5;12:678341. doi: 10.3389/fpls.2021.678341 (PMC8374242; doi:10.3389/fpls.2021.678341)
Supplement: Supplementary file 3 [file Table_1.DOCX]

**Table S1.** Results of the Kruskal-Wallis rank sum tests or ANOVA for the different water parameters with paired t-test comparison between sites that receive different levels of anthropogenic pressures in July 2019.

| Kruskal-Wallis rank sum test | **Chla ~ condition** |  |  |  |
| --- | --- | --- | --- | --- |
|  |  | chi-squared | df | p |
|  |  | 9.6211 | 1 | **0.001923** |
|  |  |  |  |  |
| Pairwise Wilcoxon rank sum test |  |  |  |  |
|  | impacted |  |  |  |
| pristine | **0.0024** |  |  |  |

| Kruskal-Wallis rank sum test | **Corg ~ condition** |  |  |  |
| --- | --- | --- | --- | --- |
|  |  | chi-squared | df | p |
|  |  | 0.89338 | 1 | 0.3446 |
|  |  |  |  |  |
| Pairwise Wilcoxon rank sum test |  |  |  |  |
|  | impacted |  |  |  |
| pristine | 0.380 |  |  |  |

| Kruskal-Wallis rank sum test | **N ~ condition** |  |  |  |
| --- | --- | --- | --- | --- |
|  |  | chi-squared | df | p |
|  |  | 0.70588 | 1 | 0.4008 |
|  |  |  |  |  |
| Pairwise Wilcoxon rank sum test |  |  |  |  |
|  | impacted |  |  |  |
| pristine | 0.440 |  |  |  |

| Kruskal-Wallis rank sum test | **C ~ condition** | |  |  |  |
| --- | --- | --- | --- | --- | --- |
|  |  | | chi-squared | df | p |
|  |  | | 1.1029 | 1 | 0.2936 |
|  |  | |  |  |  |
| Pairwise Wilcoxon rank sum test |  | |  |  |  |
|  | impacted | |  |  |  |
| pristine | 0.330 | |  |  |  |
|  | | | | | |
| ANOVA | **CN ratio ~ condition** | |  |  |  |
|  | Sum Sq | Mean Sq | F | df | p |
| CN ratio$condition | 4.69 | 4.692 | 0.548 | 1 | 0.1086 |
| Residuals | 119.88 | 8.563 |  | 14 |  |
|  |  |  |  |  |  |
| Pairwise t- test |  | |  |  |  |
|  | impacted | |  |  |  |
| pristine | 0.470 | |  |  |  |
|  | | | | | |
| Kruskal-Wallis rank sum test | **SPM ~ condition** | |  |  |  |
|  |  | | chi-squared | df | p |
|  |  | | 5.3461 | 1 | **0.02077** |
|  |  | |  |  |  |
| Pairwise Wilcoxon rank sum test |  | |  |  |  |
|  | impacted | |  |  |  |
| pristine | **0.024** | |  |  |  |
