# Supplementary material for: Nutrient History Affects the Response and Resilience of the Tropical Seagrass Halophila stipulacea to Further Enrichment in Its Native Habitat
Source: Front Plant Sci. 2021 Aug 5;12:678341. doi: 10.3389/fpls.2021.678341 (PMC8374242; doi:10.3389/fpls.2021.678341)
Supplement: Supplementary file 4 [file Table_2.DOCX]

**Table S2.** Results of the Kruskal-Wallis rank sum tests or ANOVA for the different water parameters with paired t-test comparison between sites that receive different levels of anthropogenic pressures in December 2019.

| Kruskal-Wallis rank sum test | **Corg ~ condition** |  |  |  |
| --- | --- | --- | --- | --- |
|  |  | chi-squared | df | p |
|  |  | 1.3393 | 1 | 0.2472 |
|  |  |  |  |  |
| Pairwise Wilcoxon rank sum test |  |  |  |  |
|  | impacted |  |  |  |
| pristine | 0.280 |  |  |  |

| Kruskal-Wallis rank sum test | **N ~ condition** |  |  |  |
| --- | --- | --- | --- | --- |
|  |  | chi-squared | df | p |
|  |  | 9.7634 | 1 | **0.00178** |
|  |  |  |  |  |
| Pairwise Wilcoxon rank sum test |  |  |  |  |
|  | impacted |  |  |  |
| pristine | **0.00062** |  |  |  |

| Kruskal-Wallis rank sum test | **C~ condition** |  |  |  |
| --- | --- | --- | --- | --- |
|  |  | chi-squared | df | p |
|  |  | 0.33482 | 1 | 0.5628 |
|  |  |  |  |  |
| Pairwise Wilcoxon rank sum test |  |  |  |  |
|  | impacted |  |  |  |
| pristine | 0.610 |  |  |  |

| Kruskal-Wallis rank sum test | **CN ~ condition** | |  |  |  |
| --- | --- | --- | --- | --- | --- |
|  |  | | chi-squared | df | p |
|  |  | | 10.5 | 1 | **0.001194** |
|  |  | |  |  |  |
| Pairwise Wilcoxon rank sum test |  | |  |  |  |
|  | impacted | |  |  |  |
| pristine | **0.00031** | |  |  |  |
|  | | | | | |
| ANOVA | **SPM ~ condition** | |  |  |  |
|  | Sum Sq | Mean Sq | F | df | p |
| SPM$condition | 7.206 | 7.206 | 7.149 | 1 | **0.0191** |
| Residuals | 13.104 | 1.008 |  |  |  |
|  |  | |  |  |  |
| t- test |  | |  |  |  |
|  | impacted | |  |  |  |
| pristine | **0.019** | |  |  |  |
