# Supplementary material for: Nutrient History Affects the Response and Resilience of the Tropical Seagrass Halophila stipulacea to Further Enrichment in Its Native Habitat
Source: Front Plant Sci. 2021 Aug 5;12:678341. doi: 10.3389/fpls.2021.678341 (PMC8374242; doi:10.3389/fpls.2021.678341)
Supplement: Supplementary file 5 [file Table_3.docx]

**Table S3.** Mean (± standard error (S.E.)) values of population level traits from *H. stipulacea* biomass cores in the anthropogenically-impacted (North Beach) and low impacted (South Beach) sites in control (n = 6) and fertilized (n = 6) plots. Meadow cover is expressed in percentage (%), shoot density is reported as shoots m^-2^, while biomass is in g DW m^-2^.

✱ indicates significant differences between impacted (NB) and non- impacted (SB) sites.✝ indicates significant seasonal differences between July and December, and ✢ indicates significant differences between control and fertilized plots.

| Population traits | Site | July 2019 | December 2019 | |
| --- | --- | --- | --- | --- |
|  |  |  | **Control** | **Fertilized** |
| Meadow cover | North Beach | 48.01 ± 4.34 | 77.00 ± 3.56 ✝ | 84.63 ± 4.42 |
|  | South Beach | 66.26 ± 5.58 ✱ | 64.47 ± 3.82 ✝ | 68.78 ± 6.65 |
| Shoot density | North Beach | 4053.94 ± 380.77 | 1100.07 ± 127.55 ✝ | 2458.17 ± 467.35 ✢ |
|  | South Beach | 3259.45 ± 563.75 | 1412.43 ± 405.62 ✝ | 2281.62 ± 704.12 ✢ |
| Above ground (AG) biomass | North Beach | 109.84 ± 12.51✱ | 52.45 ± 12.75 ✝ | 119.63 ± 28.14 ✱✢ |
|  | South Beach | 51.01 ± 8.13 | 20.49 ± 6.86 ✝ | 27.80 ± 9.52 ✢ |
| Below ground (BG) biomass | North Beach | 70.48 ± 7.16 | 64.20 ± 10.72 ✝ | 108.30 ± 23.33 ✱✢ |
|  | South Beach | 58.50 ± 8.63 | 26.22 ± 9.92 ✝ | 40.06 ± 9.01✢ |
| Ratio AG: BG | North Beach | 1.63 ± 0.63 ✱ | 0.90± 0.51 | 1.13 ± 0.32 |
|  | South Beach | 0.91 ± 0.10 | 0.95 ± 0.26 | 0.70 ± 0.12 |
