# Supplementary material for: Nutrient History Affects the Response and Resilience of the Tropical Seagrass Halophila stipulacea to Further Enrichment in Its Native Habitat
Source: Front Plant Sci. 2021 Aug 5;12:678341. doi: 10.3389/fpls.2021.678341 (PMC8374242; doi:10.3389/fpls.2021.678341)
Supplement: Supplementary file 6 [file Table_4.DOCX]

**Table S4.** Mean (± S.E.) values for the four subsites in SB and NB on all measured seagrass traits (population level morphological and biochemical) and sediment isotopic composition.

| Traits | Site | June 2019 | | September 2019 | |
| --- | --- | --- | --- | --- | --- |
|  |  | **Control** | **Fertilized** | **Control** | **Fertilized** |
| Population level traits | | | | | |
| Cover [%] | S1- SB | 77.68 ± 7.40 | 58.67 ± 16.41 | 58.23 ± 2.99 | 58.67 ± 10.11 |
|  | S2- SB | 61.23 ± 10.59 | 67.47 ± 11.89 | 70.70 ± 5.01 | 78.90 ± 4.07 |
|  | S3- NB | 36.67 ± 5.23 | 64.00 ± 7.77 | 83.00 ± 3.79 | 90.47 ± 2.57 |
|  | S4- NB | 36.87 ± 3.22 | 54.50 ± 6.34 | 71.00 ± 5.29 | 78.80 ± 7.56 |
| Shoot density [shoots m^-2^] | S1- SB | 2797.70 ± 718.64 | 5649.72 ± 1199.75 | 787.70 ± 313.25 | 1711.21 ± 47.05 |
|  | S2- SB | 1439.59 ± 507.43 | 3150.80 ± 391.74 | 2037.16 ± 578.11 | 2852.02 ± 1466.75 |
|  | S3- NB | 4291.61 ± 452.07 | 2797.70 ± 988.34 | 1005.00 ± 165.22 | 2335.94  ± 545.28 |
|  | S4- NB | 5242.29 ± 672.50 | 3884.18 ± 135.81 | 1195.13 ± 212.14 | 2580.40 ± 883.08 |
| AG biomass [g (DW)] | S1- SB | 31.66 ± 2.21 | 39.77 ± 7.81 | 9.41 ± 4.63 | 17.20 ± 3.27 |
|  | S2- SB | 46.00 ± 19.70 | 86.63 ± 10.07 | 31.57 ± 9.55 | 38.40 ± 18.18 |
|  | S3- NB | 106.31 ± 17.46 | 77.72 ± 28.77 | 41.56 ± 4.66 | 98.12 ± 18.93 |
|  | S4- NB | 145.23 ± 34.03 | 110.10 ± 1.56 | 63.35 ± 25.93 | 141.13 ± 56.04 |
| BG biomass [g (DW)] | S1- SB | 36.92 ± 5.26 | 39.56 ± 8.91 | 9.10 ± 0.67 | 24.44 ± 6.52 |
|  | S2- SB | 53.85 ± 6.05 | 103.66 ± 7.31 | 43.33 ± 14.09 | 55.69 ± 10.91 |
|  | S3- NB | 92.68 ± 21.44 | 50.46 ± 8.82 | 80.60 ± 15.32 | 100.02 ± 40.48 |
|  | S4- NB | 68.04 ± 4.80 | 70.73 ± 10.78 | 47.81 ± 8.39 | 116.57 ± 31.86 |
| AG:BG | S1- SB | 0.90 ± 0.16 | 1.03 ± 0.05 | 1.11 ± 0.55 | 0.74 ± 0.12 |
|  | S2- SB | 0.85 ± 0.37 | 0.85 ± 0.14 | 0.80 ± 0.15 | 0.66 ± 0.22 |
|  | S3- NB | 1.31 ± 0.40 | 1.44 ± 0.37 | 0.58 ± 0.17 | 1.14 ± 0.20 |
|  | S4- NB | 2.10 ± 0.38 | 1.65 ± 0.32 | 1.23 ± 0.29 | 1.14 ± 0.21 |
| Morphological traits | | | | | |
| No. of leaves [per m²] | S1- SB | 7225.12 ± 1868.47 | 15373.75 ± 3059.57 | 2064.32 ± 889.33 | 4345.94 ± 287.46 |
|  | S2- SB | 3531.07 ± 1263.55 | 7877.01 ± 961.09 | 5052.15 ± 1493.67 | 6899.17 ± 3398.20 |
|  | S3- NB | 9452.41 ± 954.93 | 6518.91 ± 2352.31 | 2444.59 ± 376.37 | 5269.45 ± 1228.02 |
|  | S4- NB | 12059.97 ± 1478.78 | 8691.87 ± 391.74 | 2987.83 ± 518.22 | 5921.34 ± 1868.47 |
| Leaf height [cm] | S1- SB | 2.61 ± 0.09 | 2.42 ± 0.08 | 2.59 ± 0.13 | 2.70 ± 0.10 |
|  | S2- SB | 3.07 ± 0.15 | 3.28 ± 0.12 | 3.04 ± 0.11 | 2.98 ± 0.10 |
|  | S3- NB | 4.08 ± 0.08 | 4.27 ± 0.11 | 3.92 ± 0.11 | 4.56 ± 0.11 |
|  | S4- NB | 4.44 ± 0.10 | 4.52 ± 0.10 | 4.35 ± 0.24 | 4.79 ± 0.11 |
| Apical shoots [%] | S1- SB | 29.01 ± 0.24 | 36.65 ± 5.04 | 21.98 ± 12.38 | 26.72 ± 4.94 |
|  | S2- SB | 22.14 ± 1.07 | 25.12 ± 1.71 | 23.13 ± 2.20 | 23.75 ± 5.78 |
|  | S3- NB | 10.23 ± 0.57 | 15.95 ± 1.16 | 21.99 ± 1.81 | 12.71 ± 1.10 |
|  | S4- NB | 15.18 ± 0.77 | 11.82 ± 1.58 | 25.44 ± 2.92 | 17.50 ± 4.65 |
| LAI [m² leaf area per shoot * shoot density per m²] | S1- SB | 1.15 ± 0.07 | 1.03 ± 0.06 | 1.19 ± 0.09 | 3.91 ± 0.74 |
|  | S2- SB | 1.55 ± 0.15 | 1.76 ± 0.11 | 1.60 ± 0.10 | 1.59 ± 0.09 |
|  | S3- NB | 2.86 ± 0.09 | 3.16 ± 0.11 | 2.81 ± 0.14 | 3.65 ± 0.13 |
|  | S4- NB | 3.41 ± 0.13 | 3.75 ± 0.11 | 3.58 ± 0.30 | 4.16 ± 0.18 |
| Leaf width [cm] | S1- SB | 0.72 ± 0.04 | 0.75 ± 0.06 | 0.66 ± 0.04 | 0.50 ± 0.05 |
|  | S2- SB | 0.68 ± 0.03 | 0.77 ± 0.04 | 0.75 ± 0.03 | 0.74 ± 0.03 |
|  | S3- NB | 0.95 ± 0.03 | 1.12 ± 0.05 | 1.06 ± 0.06 | 1.28 ± 0.04 |
|  | S4- NB | 1.07 ± 0.03 | 1.08 ± 0.02 | 1.11 ± 0.06 | 1.20 ± 0.05 |
| Leaf area [cm] | S1- SB | 1.02 ± 0.06 | 0.91 ± 0.05 | 1.06 ± 0.08 | 3.47 ± 0.65 |
|  | S2- SB | 1.38 ± 0.14 | 1.57 ± 0.10 | 1.42 ± 0.10 | 1.41 ± 0.08 |
|  | S3- NB | 2.55 ± 0.08 | 2.81 ± 0.10 | 2.50 ± 0.12 | 3.24 ± 0.12 |
|  | S4- NB | 3.03 ± 0.12 | 3.33 ± 0.10 | 3.19 ± 0.27 | 3.70 ± 0.16 |
| Internodal distances [mm] | S1- SB | 9.67 ± 0.36 | 8.22 ± 0.31 | 9.87 ± 0.64 | 8.52 ± 0.54 |
|  | S2- SB | 7.34 ± 0.38 | 8.61 ± 0.35 | 9.09 ± 0.37 | 9.46 ± 0.48 |
|  | S3- NB | 10.22 ± 0.35 | 10.17 ± 0.15 | 11.17 ± 2.26 | 9.00 ± 0.29 |
|  | S4- NB | 10.69 ± 0.28 | 10.71 ± 0.26 | 8.03 ± 0.29 | 10.31 ± 0.34 |
| Biochemical traits | | | | | |
| Leaf C [% DW] | S1- SB | 24.02 ± 0.66 | 25.26 ± 1.61 | 24.58 ± 3.72 | 22.11 ± 0.77 |
|  | S2- SB | 25.00 ± 0.42 | 24.76 ± 0.30 | 17.78 ± 2.23 | 25.78 ± 4.19 |
|  | S3- NB | 22.30 ± 0.79 | 21.77 ± 1.22 | 20.80 ± 0.14 | 20.26 ± 0.64 |
|  | S4- NB | 25.15 ± 0.26 | 25.17 ± 0.42 | 19.89 ± 0.48 | 20.68 ± 0.61 |
| Rhizome C [% DW] | S1- SB | 24.30 ± 2.86 | 25.76 ± 0.50 | 27.29 ± 5.86 | 20.70 ± 0.87 |
|  | S2- SB | 27.19 ± 0.42 | 26.88 ± 0.46 | 25.44 ± 1.60 | 20.67 ± 0.21 |
|  | S3- NB | 26.11 ± 0.13 | 27.09 ± 0.21 | 25.54 ± 0.46 | 24.30 ± 2.06 |
|  | S4- NB | 27.07 ± 0.45 | 28.34 ± 0.46 | 24.08 ± 0.67 | 24.44 ± 0.35 |
| Leaf N [% DW] | S1- SB | 0.90 ± 0.05 | 1.02 ± 0.08 | 1.43 ± 0.36 | 1.08 ± 0.06 |
|  | S2- SB | 1.14 ± 0.07 | 1.07 ± 0.03 | 0.77 ± 0.03 | 1.46 ± 0.35 |
|  | S3- NB | 1.00 ± 0.05 | 1.05 ± 0.07 | 1.04 ± 0.07 | 1.20 ± 0.07 |
|  | S4- NB | 1.01 ± 0.02 | 1.05 ± 0.02 | 1.00 ± 0.03 | 1.19 ± 0.07 |
| Rhizome N [% DW] | S1- SB | 0.30 ± 0.07 | 0.34 ± 0.02 | 1.40 ± 1.02 | 0.66 ± 0.08 |
|  | S2- SB | 0.35 ± 0.03 | 0.42 ± 0.02 | 0.47 ± 0.06 | 0.62 ± 0.15 |
|  | S3- NB | 0.48 ± 0.01 | 0.60 ± 0.08 | 0.81 ± 0.20 | 1.23 ± 0.14 |
|  | S4- NB | 0.55 ± 0.02 | 0.50 ± 0.07 | 1.09 ± 0.09 | 1.38 ± 0.34 |
| Leaf δ13C [‰] | S1- SB | - 7.12 ± 0.13 | - 7.31± 0.09 | - 9.58 ± 1.72 | - 9.79 ± 1.57 |
|  | S2- SB | - 6.33 ± 0.12 | - 6.64 ± 0.12 | - 6.82 ± 0.17 | - 9.36 ± 2.04 |
|  | S3- NB | - 6.70 ± 0.29 | - 7.32 ± 0.10 | - 7.24 ± 0.15 | - 7.82 ± 0.50 |
|  | S4- NB | - 6.74 ± 0.13 | - 6.58 ± 0.13 | - 8.51 ± 0.16 | - 8.33 ± 0.34 |
| Rhizome δ13C [‰] | S1- SB | - 7.49 ± 0.22 | - 7.98 ± 0.09 | - 9.56 ± 1.84 | - 7.89 ± 0.15 |
|  | S2- SB | - 6.90 ± 0.08 | - 7.00 ± 0.31 | - 7.46 ± 0.25 | - 7.43 ± 0.13 |
|  | S3- NB | - 6.86 ± 0.16 | - 7.80 ± 0.21 | - 7.18 ± 0.08 | - 7.85 ± 0.70 |
|  | S4- NB | - 7.43 ± 0.13 | - 7.45 ± 0.18 | - 8.27 ± 0.30 | - 8.54 ± 0.10 |
| Leaf δ15N [‰] | S1- SB | 1.29 ± 0.17 | 1.37 ± 0.15 | 0.61 ± 2.05 | 0.59 ± 2.29 |
|  | S2- SB | 1.83 ± 0.38 | 0.99 ± 0.55 | 0.04 ± 0.20 | - 1.63 ± 3.02 |
|  | S3- NB | 1.22 ± 0.20 | 1.35 ± 0.39 | 0.34 ± 0.08 | - 2.82 ± 0.17 |
|  | S4- NB | 2.89 ± 0.12 | 2.89 ± 0.04 | 1.98 ± 0.17 | - 1.95 ± 0.75 |
| Rhizome δ15N [‰] | S1- SB | 0.61 ± 0.22 | 0.52 ± 0.23 | 1.09 ± 2.52 | - 4.65 ± 0.66 |
|  | S2- SB | 1.35 ± 0.31 | 0.52 ± 0.38 | 0.28 ± 0.37 | - 4.60 ± 0.88 |
|  | S3- NB | 0.59 ± 0.10 | 0.47 ± 0.06 | - 0.24 ± 0.14 | - 3.90 ± 0.81 |
|  | S4- NB | 2.18 ± 0.21 | 1.90 ± 0.09 | 1.40 ± 0.20 | - 4.40 ± 0.59 |
| Leaf C:N ratio | S1- SB | 26.68 ± 0.88 | 24.87 ± 0.88 | 18.08 ± 1.67 | 20.48 ± 0.45 |
|  | S2- SB | 22.00 ± 1.16 | 23.12 ± 0.47 | 22.90 ± 2.11 | 18.51 ± 2.12 |
|  | S3- NB | 22.33 ± 0.43 | 20.72 ± 0.50 | 20.22 ± 1.43 | 16.98 ± 0.74 |
|  | S4- NB | 24.99 ± 0.19 | 23.91 ± 0.60 | 19.83 ± 0.62 | 17.52 ± 1.01 |
| Rhizome C:N ratio | S1- SB | 78.50 ± 9.80 | 76.72 ± 5.02 | 43.01 ± 17.51 | 32.82 ± 5.56 |
|  | S2- SB | 80.25 ± 8.29 | 63.51 ± 1.67 | 56.08 ± 5.60 | 37.32 ± 7.52 |
|  | S3- NB | 54.75 ± 1.25 | 46.60 ± 6.36 | 35.86 ± 7.29 | 20.51 ± 3.97 |
|  | S4- NB | 49.18 ± 0.74 | 59.83 ± 9.35 | 22.52 ± 2.52 | 20.75 ± 6.22 |
| Leaf P [µg g^-1^] | S1- SB | 1128.45 ± 360.92 | 1310.92 ± 219.03 | 1382.36 ± 349.87 | 1022.45 ± 149.31 |
|  | S2- SB | 1190.80 ± 378.87 | 1056.54 ± 217.42 | 1343.28 ± 468.49 | 1339.83 ± 170.72 |
|  | S3- NB | 1225.87 ± 276.30 | 1581.74 ± 257.67 | 1595.65 ± 153.66 | 1429.79 ± 142.96 |
|  | S4- NB | 1367.42 ± 35.34 | 1823.07 ± 381.20 | 2406.29 ± 132.39 | 1169.08 ± 112.44 |
| Rhizome P [µg g^-1^] | S1- SB | 855.71 ± 127.88 | 948.03 ± 134.76 | 630.97 ± 179.16 | 665.00 ± 206.76 |
|  | S2- SB | 1021.00 ± 123.99 | 1121.34 ± 192.28 | 956.66 ± 167.59 | 939.62 ± 366.62 |
|  | S3- NB | 1562.56 ± 72.70 | 1666.50 ± 158.73 | 1462.00 ± 155.65 | 1675.59 ± 277.08 |
|  | S4- NB | 1794.58 ± 75.08 | 1792.51 ± 19.65 | 2246.31 ± 201.17 | 2000.38 ± 307.72 |
| Starch in the rhizome [sucrose eq mg g (DW)^-1^] | S1- SB | 72.05 ± 12.10 | 76.33 ± 4.56 | 85.00 ± 28.74 | 63.94 ± 3.04 |
|  | S2- SB | 87.28 ± 10.16 | 85.58 ± 1.78 | 81.11 ± 15.88 | 73.47 ± 8.16 |
|  | S3- NB | 82.61 ± 10.53 | 75.26 ± 10.23 | 54.29 ± 2.55 | 52.81 ± 2.68 |
|  | S4- NB | 63.36 ± 4.16 | 76.62 ± 13.19 | 34.53 ± 2.57 | 45.41 ± 9.23 |
| Sugar in the rhizome [sucrose eq mg g (DW)^-1^] | S1- SB | 94.43 ± 14.14 | 118.58 ± 22.38 | 58.26 ± 14.48 | 60.00 ± 13.22 |
|  | S2- SB | 138.00 ± 12.07 | 104.27 ± 11.38 | 161.27 ± 25.02 | 84.22 ± 34.91 |
|  | S3- NB | 103.08 ± 13.15 | 121.10 ± 13.00 | 117.00 ± 22.72 | 51.55 ± 29.27 |
|  | S4- NB | 116.89 ± 12.88 | 143.87 ± 21.76 | 59.66 ± 21.74 | 58.82 ± 11.77 |
| Starch in the leaf [sucrose eq mg g (DW)^-1^] | S1- SB | 99.02 ± 10.83 | 105.31 ± 4.66 | 106.45 ± 46.38 | 63.32 ± 2.30 |
|  | S2- SB | 81.92 ± 11.11 | 67.61 ± 12.13 | 59.05 ± 3.08 | 60.84 ± 6.94 |
|  | S3- NB | 54.41 ± 5.14 | 53.05 ± 5.04 | 48.75 ± 6.86 | 43.43 ± 5.13 |
|  | S4- NB | 62.06 ± 11.85 | 56.33 ± 9.58 | 39.88 ± 4.18 | 38.54 ± 3.53 |
| Sugar in the leaf [sucrose eq mg g (DW)^-1^] | S1- SB | 65.14 ± 12.41 | 65.08 ± 8.83 | 38.03 ± 6.18 | 26.00 ± 1.84 |
|  | S2- SB | 38.00 ± 5.51 | 41.12 ± 4.62 | 37.31 ± 7.13 | 41.51 ± 1.17 |
|  | S3- NB | 25.12 ± 2.87 | 28.43 ± 1.66 | 24.00 ± 2.87 | 22.96 ± 3.59 |
|  | S4- NB | 17.15 ± 0.36 | 28.30 ± 4.04 | 21.89 ± 1.40 | 23.33 ± 1.63 |
| Isotopic signatures in the underlying sediment | | | | | |
| δ^15^N [‰] | S1- SB | 1.66 ± 0.80 | 1.31 ± 0.43 | 1.58 ± 0.25 | 0.24 ± 0.43 |
|  | S2- SB | 1.88 ± 0.38 | 2.55 ± 0.77 | 0.58 ± 0.58 | 1.42 ± 0.73 |
|  | S3- NB | 2.82 ± 0.56 | 2.11 ± 0.18 | 2.46 ± 0.18 | 2.42 ± 0.10 |
|  | S4- NB | 4.55 ± 0.13 | 4.22 ± 0.26 | 4.40 ± 0.23 | 4.60 ± 0.10 |
| δ^13^C [‰] | S1- SB | - 15.22 ± 0.29 | - 14.69 ± 0.24 | - 14.39 ± 0.20 | - 14.35 ± 0.35 |
|  | S2- SB | - 14.29 ± 0.55 | - 14.89 ± 0.34 | - 14.51 ± 0.17 | - 16.07 ± 0.47 |
|  | S3- NB | - 15.75 ± 0.54 | - 14.86 ± 0.21 | - 14.58 ± 0.09 | - 14.89 ± 0.27 |
|  | S4- NB | - 16.84 ± 0.21 | - 16.81 ± 0.24 | - 15.67 ± 0.17 | - 15.18 ± 0.16 |
