# Supplementary material for: Nutrient History Affects the Response and Resilience of the Tropical Seagrass Halophila stipulacea to Further Enrichment in Its Native Habitat
Source: Front Plant Sci. 2021 Aug 5;12:678341. doi: 10.3389/fpls.2021.678341 (PMC8374242; doi:10.3389/fpls.2021.678341)
Supplement: Supplementary file 7 [file Table_5.DOCX]

**Table S5.** Linear mixed effect model (LME) selection for natural stable isotope content (δ^15^N and δ^13^C) in sediments underlying *H. stipulacea* meadows to condition (high or low impacted site) and treatment (fertilization and control). df = degrees of freedom. AICc = Akaike Information Criterion corrected for small sample sizes. ΔAICc = difference AICc values between each model and the best fitting model with the lowest AICc. AICcWt = Akaike weights. LL= Likelihood. The significance of each independent variable (or interaction) in each model was assessed using the likelihood ratio (LR) test by comparing models with the variable of interest against the null or reduced model (Winter 2013). Significant parameters are in bold.

| Model ranking | Model | df | AICc | ΔAICc | AICcWt | LL | χ2 | p value | R² |
| --- | --- | --- | --- | --- | --- | --- | --- | --- | --- |
| **δ^13^C - Sediment** | | | | | | | | | |
| June 2019 | | | | | | | | | |
| 1 | δ^13^C ~ condition | 4 | 61.3 | 0.0 | 0.428 | -25.55 | 3.44 | 0.0636 | 0.641 |
| 2 | Intercept only (δ^13^C ~ 1) | 3 | 61.8 | 0.5 | 0.333 | -27.28 |  |  |  |
| 3 | δ^13^C ~ condition + treatment | 5 | 64.0 | 2.7 | 0.109 | -25.26 |  |  |  |
| 4 | δ^13^C ~ treatment | 4 | 64.2 | 2.9 | 0.102 | -26.98 | 0.58 | 0.4469 |  |
| 5 | δ^13^C ~ condition + treatment + interaction | 6 | 66.8 | 5.5 | 0.027 | -24.80 | 0.92 | 0.3383 |  |
| September 2019 | | | | | | | | | |
| 1 | Intercept only (δ^13^C ~ 1) | 3 | 55.8 | 0.0 | 0.462 | -24.32 |  |  | 0.368 |
| 2 | δ^13^C ~ treatment | 4 | 56.8 | 1.0 | 0.281 | -23.37 | 1.91 | 0.1673 |  |
| 3 | δ^13^C ~ condition | 4 | 58.4 | 2.5 | 0.129 | -24.14 | 0.36 | 0.5511 |  |
| 4 | δ^13^C ~ condition + treatment | 5 | 59.7 | 3.9 | 0.067 | -23.19 |  |  |  |
| 5 | δ^13^C ~ condition + treatment + interaction | 6 | 59.9 | 4.1 | 0.061 | -21.48 | 3.42 | 0.0645 |  |
| **δ^15^N - Sediment** | | | | | | | | | |
| June 2019 | | | | | | | | | |
| **1** | Intercept only (δ^15^N ~ 1) | 3 | **33.5** | **0.0** | **0.672** | **-11.69** | **6.14** | **0.0132** | **0.521** |
| 2 | δ^15^N ~ condition | 4 | 36.7 | 3.2 | 0.135 | -11.67 |  |  |  |
| 3 | δ^15^N ~ treatment | 4 | 36.7 | 3.2 | 0.133 | -14.76 |  |  |  |
| 4 | δ^15^N ~ condition + treatment | 5 | 39.6 | 6.1 | 0.032 | -14.75 | 0.02 | 0.8781 |  |
| 5 | δ^15^N ~ condition + treatment + interaction | 6 | 39.9 | 6.4 | 0.027 | -11.47 | 0.41 | 0.5210 |  |
| September 2019 | | | | | | | | | |
| **1** | **δ^15^N ~ treatment** | **4** | **85.5** | **0.0** | **0.639** | **-39.15** | **0.05** | **0.8240** | **0.199** |
| 2 | Intercept only (δ^15^N ~ 1) | 3 | 88.3 | 2.8 | 0.161 | -39.08 |  |  |  |
| 3 | δ^15^N ~ condition + treatment | 5 | 88.4 | 2.9 | 0.153 | -39.13 |  |  |  |
| 4 | δ^15^N ~ condition | 4 | 91.4 | 5.9 | 0.033 | -39.05 | 0.15 | 0.6998 |  |
| 5 | δ^15^N ~ condition + treatment + interaction | 6 | 93.2 | 7.7 | 0.014 | -38.12 | 1.86 | 0.1721 |  |
| **Differences over time (July 2019 to December 2019)** | | | | | | | | | |
| **δ^13^C – Sediment** | | | | | | | | | |
| **1** | **δ^13^C ~ time** | **4** | **59.7** | **0.0** | **0.852** | **-24.75** | **6.45** | **0.0111** | **0.630** |
| 2 | Intercept only (δ^13^C ~ 1) | 3 | 63.2 | 3.5 | 0.148 | -27.98 |  |  |  |
| **δ^15^N – Sediment** | | | | | | | | | |
| 1 | Intercept only (δ^15^N ~ 1) | 3 | 72.9 | 0.0 | 0.603 | -32.83 | 2.13 | 0.1449 | 0.732 |
| 2 | δ^15^N ~ time | 4 | 73.8 | 0.8 | 0.397 | -31.77 |  |  |  |
