# Supplementary material for: Nutrient History Affects the Response and Resilience of the Tropical Seagrass Halophila stipulacea to Further Enrichment in Its Native Habitat
Source: Front Plant Sci. 2021 Aug 5;12:678341. doi: 10.3389/fpls.2021.678341 (PMC8374242; doi:10.3389/fpls.2021.678341)
Supplement: Supplementary file 9 [file Table_7.docx]

**Table S7.** Linear mixed effect model (LME) selection for population level responses of *H. stipulacea* over time (July 2019 to December 2019). df = degrees of freedom. AICc = Akaike Information Criterion corrected for small sample sizes. ΔAICc = difference AICc values between each model and the best fitting model with the lowest AICc. AICcWt = Akaike weights. LL= Likelihood. The significance of time was assessed using the likelihood ratio (LR) test by comparing models with the time added against the null model.

| Model ranking | Model | df | AICc | ΔAICc | AICcWt | LL | χ2 | p value | R² |
| --- | --- | --- | --- | --- | --- | --- | --- | --- | --- |
| Cover | | | | | | | | | |
| **1** | **Cover ~ time** | **4** | **211.6** | **0.0** | **0.845** | **-100.74** | **6.29** | **0.0121** | **0.231** |
| 2 | Intercept only (Cover ~ 1) | 3 | 215.0 | 3.4 | 0.155 | -103.89 |  |  |  |
| Shoot density | | | | | | | | | |
| **1** | **Sdens ~ time** | **4** | **415.0** | **0.0** | **0.983** | **-202.45** | **10.982** | **0.0009** | **0.4209** |
| 2 | Intercept only (Sdens ~ 1) | 3 | 423.1 | 8.1 | 0.017 | -207.94 |  |  |  |
| Aboveground (AG) biomass | | | | | | | | | |
| **1** | **AG ~ time** | **4** | **252.1** | **0.0** | **0.964** | **-120.99** | **9.48** | **0.0021** | **0.639** |
| 2 | Intercept only (AG ~ 1) | 3 | 258.7 | 6.6 | 0.036 | -125.73 |  |  |  |
| Belowground (BG) biomass | | | | | | | | | |
| **1** | **BG ~ time** | **4** | **226.3** | **0.0** | **-108.12** | **0.74** | **5.00** | **0.0254** | **0.683** |
| 2 | Intercept only (BG ~ 1) | 3 | 228.4 | 2.1 | -110.62 | 1.00 |  |  |  |
| AG : BG ratio | | | | | | | | | |
| 1 | Intercept only (AG : BG ~ 1) | 3 | 53.8 | 0.0 | 0.580 | -23.30 | 2.26 | 0.1328 | 0.257 |
| 2 | AG : BG ~ time | 4 | 54.5 | 0.6 | 0.420 | -22.17 |  |  |  |
