# Supplementary material for: Nutrient History Affects the Response and Resilience of the Tropical Seagrass Halophila stipulacea to Further Enrichment in Its Native Habitat
Source: Front Plant Sci. 2021 Aug 5;12:678341. doi: 10.3389/fpls.2021.678341 (PMC8374242; doi:10.3389/fpls.2021.678341)
Supplement: Supplementary file 11 [file Table_9.DOCX]

**Table S9.** Linear mixed effect model (LME) selection for morphological responses of *H. stipulacea* over time (July 2019 to December 2019). df = degrees of freedom. AICc = Akaike Information Criterion corrected for small sample sizes. ΔAICc = difference AICc values between each model and the best fitting model with the lowest AICc. AICcWt = Akaike weights. LL= Likelihood. The significance of time was assessed using the likelihood ratio (LR) test by comparing models with the time added against the null model.

| Model ranking | Model | df | AICc | ΔAICc | AICcWt | LL | χ2 | p value | R² |
| --- | --- | --- | --- | --- | --- | --- | --- | --- | --- |
| Number of leaves | | | | | | | | | |
| **1** | **lvs~ time** | **4** | **457,0** | **0,0** | **0,974** | **-223,44** | **10.21** | **0.0015** | **0.418** |
| 2 | Intercept only (lvs ~ 1) | 3 | 464,2 | 7,2 | 0,026 | -228,50 |  |  |  |
| Leaf length | | | | | | | | | |
| 1 | Intercept only (height ~ 1) | 3 | 443,5 | 0,0 | 0,661 | -218,71 | 0,74 | 0.3886 | 0.562 |
| 2 | height ~ time | 4 | 444,9 | 1,3 | 0,339 | -218,34 |  |  |  |
| Leaf width | | | | | | | | | |
| 1 | Intercept only (width ~ 1) | 3 | -18,1 | 0,0 | 0,562 | 12,08 | 1.57 | 0.2096 | 0.378 |
| 2 | width ~ time | 4 | -17,6 | 0,5 | 0,438 | 12,87 |  |  |  |
| Internodal distances | | | | | | | | | |
| **1** | **nod ~ time** | **4** | **-30,3** | **0,0** | **0,733** | **19,23** | **4.08** | **0.0434** | **0.084** |
| 2 | Intercept only (nod ~ 1) | 3 | -28,3 | 2,0 | 0,267 | 17,19 |  |  |  |
| Percent apical shoots | | | | | | | | | |
| 1 | Intercept only (papical ~ 1) | 3 | 178,3 | 0,0 | 0,685 | -85,57 | 1.35 | 0.2456 | 0.161 |
| 2 | papical ~ time | 4 | 179,9 | 1,6 | 0,315 | -84,90 |  |  |  |
| Leaf area | | | | | | | | | |
| 1 | Intercept only (l_area ~ 1) | 3 | 441,8 | 0,0 | 0,707 | -217,84 | 0.31 | 0.5779 | 0.550 |
| 2 | l_area ~ time | 4 | 443,6 | 1,8 | 0,293 | -217,68 |  |  |  |
| LAI | | | | | | | | | |
| 1 | Intercept only (LAI ~ 1) | 3 | 493,0 | 0,0 | 0,707 | -243,42 | 0.31 | 0.5779 | 0.550 |
| 2 | LAI ~ time | 4 | 494,7 | 1,8 | 0,293 | -243,27 |  |  |  |
