# Supplementary material for: Nutrient History Affects the Response and Resilience of the Tropical Seagrass Halophila stipulacea to Further Enrichment in Its Native Habitat
Source: Front Plant Sci. 2021 Aug 5;12:678341. doi: 10.3389/fpls.2021.678341 (PMC8374242; doi:10.3389/fpls.2021.678341)
Supplement: Supplementary file 13 [file Table_11.DOCX]

**Table S11.** Linear mixed effect model (LME) selection for nutrient content in leaves and rhizomes of *H. stipulacea* over time (July 2019 to December 2019). df = degrees of freedom. AICc = Akaike Information Criterion corrected for small sample sizes. ΔAICc = difference AICc values between each model and the best fitting model with the lowest AICc. AICcWt = Akaike weights. LL= Likelihood. The significance of time was assessed using the likelihood ratio (LR) test by comparing models with the time added against the null model.

| Model ranking | Model | df | AICc | ΔAICc | AICcWt | LL | χ2 | p value | R² |
| --- | --- | --- | --- | --- | --- | --- | --- | --- | --- |
| CN ratio- Leaves | | | | | | | | | |
| **1** | **cnleaves ~ time** | **4** | **122.6** | **0.0** | **0.978** | **-56.27** | **10.53** | **0.0012** | **0.355** |
| 2 | Intercept only (cnleaves ~ 1) | 3 | 130.3 | 7.6 | 0.022 | -61.53 |  |  |  |
| Carbon content - Leaves | | | | | | | | | |
| **1** | **C ~ time** | **4** | **130.3** | **0.0** | **0.869** | **-60.10** | **6.69** | **0.0097** | **0.116** |
| 2 | Intercept only (C ~ 1) | 3 | 134.1 | 3.8 | 0.131 | -63.44 |  |  |  |
| Nitrogen content - Leaves | | | | | | | | | |
| 1 | Intercept only (N ~ 1) | 3 | 11.1 | 0.0 | 0.795 | -1.96 | 0.19 | 0.6621 | 0.087 |
| 2 | N ~ time | 4 | 13.8 | 2.7 | 0.205 | -1.86 |  |  |  |
| Phosphorous content - Leaves | | | | | | | | | |
| 1 | Intercept only (P ~ 1) | 3 | 366.3 | 0.0 | 0.707 | -179.53 | 1.14 | 0.2854 | 0.145 |
| 2 | P ~ time | 4 | 368.0 | 1.8 | 0.293 | -178.96 |  |  |  |
| CN ratio- Rhizomes | | | | | | | | | |
| **1** | **cnrhizomes~ time** | **4** | **209.5** | **0.0** | **0.998** | **-99.68** | **15.62** | **0.0001** | **0.688** |
| 2 | Intercept only (cnrhizomes ~ 1) | 3 | 222.2 | 12.7 | 0.002 | -107.49 |  |  |  |
| Carbon content- Rhizomes | | | | | | | | | |
| 1 | Intercept only (C ~ 1) | 3 | 136.7 | 0.0 | 0.798 | -64.76 | 0.16 | 0.6884 | 0.006 |
| 2 | C ~ time | 4 | 139.5 | 2.7 | 0.202 | -64.68 |  |  |  |
| Nitrogen content- Rhizomes | | | | | | | | | |
| **1** | **N~ time** | **4** | **52.0** | **0.0** | **0.673** | **-20.94** | **4.35** | **0.0371** | **0.166** |
| 2 | Intercept only (N~ 1) | 3 | 53.4 | 1.4 | 0.327 | -23.11 |  |  |  |
| Phosphorous content- Rhizomes | | | | | | | | | |
| 1 | Intercept only (P ~ 1) | 3 | 355.3 | 0.0 | 0.809 | -174.05 | 0.02 | 0.8856 | 0.805 |
| 2 | P ~ time | 4 | 358.2 | 2.9 | 0.191 | -174.04 |  |  |  |
